# Synergistic effects of complex drug combinations in colorectal cancer cells predicted by logical modelling

**DOI:** 10.3389/fsysb.2023.1112831

**Published:** 2023-02-27

**Authors:** Evelina Folkesson, B. Cristoffer Sakshaug, Andrea D. Hoel, Geir Klinkenberg, Åsmund Flobak

**Affiliations:** ^1^ Department of Clinical and Molecular Medicine, Norwegian University of Science and Technology, Trondheim, Norway; ^2^ Department of Biotechnology, SINTEF Materials and Chemistry, Trondheim, Norway; ^3^ The Cancer Clinic, St Olav’s University Hospital, Trondheim, Norway

**Keywords:** colorectal cancer, logical modelling, drug combinations, synergy, prediction

## Abstract

Drug combinations have been proposed to combat drug resistance in cancer, but due to the large number of possible drug targets, *in vitro* testing of all possible combinations of drugs is challenging. Computational models of a disease hold great promise as tools for prediction of response to treatment, and here we constructed a logical model integrating signaling pathways frequently dysregulated in cancer, as well as pathways activated upon DNA damage, to study the effect of clinically relevant drug combinations. By fitting the model to a dataset of pairwise combinations of drugs targeting MEK, PI3K, and TAK1, as well as several clinically approved agents (palbociclib, olaparib, oxaliplatin, and 5FU), we were able to perform model simulations that allowed us to predict more complex drug combinations, encompassing sets of three and four drugs, with potentially stronger effects compared to pairwise drug combinations. All predicted third-order synergies, as well as a subset of non-synergies, were successfully confirmed by *in vitro* experiments in the colorectal cancer cell line HCT-116, highlighting the strength of using computational strategies to rationalize drug testing.

## Introduction

Despite the fact that cancer is characterized by excessive proliferation, chemotherapies and other therapies aimed at broadly targeting proliferating cells have had limited efficacy as treatments of cancer (Marin et al., 2009; Munker et al., 2018). Targeted therapy, which is treatment of cancer using drugs that specifically target proteins that play a role in the growth control signaling network, has in several cases proven advantageous over chemotherapies, by demonstrating improved efficacy, as well as reduced side-effects (Jonker et al., 2007; Price et al., 2014; Fakih, 2015). Especially, specific molecular targeted agents have shown significant effect in certain patient subpopulations, such as the effect of EGFR inhibitors in EGFR-mutated non-small-cell lung cancers (NSCLC) (Solassol et al., 2019), BRAF inhibitors in advanced BRAF V600-mutated melanoma (Kim et al., 2014), and PARP inhibitors in cancer patients carrying BRCA mutations (Fong et al., 2009). Despite this progress, the widespread use of targeted therapies is still limited, for several reasons. On one hand, molecular heterogeneity among patients makes accurate patient stratification essential for successful use of molecularly targeted agents. This process has for long been hampered by the relatively small number of biomarkers validated for response prediction. On the other hand, pathway crosstalk, or rewiring of signaling pathways, often leads to drug resistance and relapse of disease (Ellis and Hicklin, 2009; Ahronian et al., 2015; Liu et al., 2018). While the advancement of ‘omics technologies has led to progress within biomarker discovery (Li et al., 2019; Srivastava and Creek, 2019), the use of combination therapy, i.e., targeting of cancer signaling networks at multiple points, was demonstrated to be a promising strategy for overcoming obstacles related to inherent or acquired drug resistance. The use of drug combinations to increase the effect of treatment is supported by multiple successes from *in vitro* and clinical cancer research, with dual inhibition of MAPK-ERK pathway components (Khunger et al., 2018), and combined inhibition of MEK and components of the PI3K signaling pathway being some of the most prominent examples (Jokinen and Koivunen, 2015; Arend et al., 2020). Of these, combined inhibition of BRAF and MEK is, in addition, clinically used for treatment of BRAF V600-mutated NSCLC and melanoma(Eroglu and Ribas, 2016; Han et al., 2021). So far, low- and high-throughput drug screens, as well as more hypothesis-driven investigations have only identified a small number of synergistic drug combinations. This is in part due to the enormous combinatorial space that needs to be explored for synergistic combinations to be detected (Amzallag et al., 2019). The size and complexity of cell signaling networks call for the identification of synergistic drug combinations using various kinds of computational models. Such models have been found predictive of treatment response to both single and combined cancer treatment (Sahin et al., 2009; Grieco et al., 2013; Flobak et al., 2015; Saez-Rodriguez et al., 2015; Garland, 2017; AstraZeneca-Sanger Drug Combination DREAM Consortium et al., 2019; Niederdorfer et al., 2020). However, despite the even greater need for computational assistance in predicting response to more complex drug combinations, i.e., more than two drugs together, few such studies have been conducted (Geva-Zatorsky et al., 2010; Zimmer et al., 2016; 2017; Katzir et al., 2019). In light of studies revealing cell line-specific resistance to pairwise drug combinations (Tomska et al., 2018; Folkesson et al., 2020), we believe that computational models predictive of responses to higher-order drug combinations (>2 drugs) will fill an important gap in computational medicine. In this study, we present a logical model encompassing signaling pathways frequently dysregulated in cancer, as well as pathways activated upon DNA damage and repair. The model is intended for usage within studies of combination effects of second- and higher-order combinations involving drugs targeting MAPK, PI3K and TAK1/NF-kB signaling pathways, as well as the clinically approved agents palbociclib (CDK4/6 inhibitor), olaparib (PARP inhibitor), oxaliplatin and 5FU (chemotherapeutic agents). The model was constructed in the software GINsim and focuses on the targets of drugs tested in our previously published high-throughput combination screen (Folkesson et al., 2020). Following model adjustments guided by drug response data of pairwise combination treatment, we demonstrate the capacity of the model to predict synergistic effects of three-way and four-way (third- and fourth-order) drug combinations. All higher-order combinations classified *in silico* as synergistic, as well as a subset of predicted non-synergies, were successfully confirmed in a novel *in vitro* screen investigating the effect of higher-order combinations in the colorectal cancer (CRC) line HCT-116. Altogether, our results highlight the advantage of using computational strategies for rationalization of drug testing *in vitro*.

## Materials and methods

### Construction of the regulatory network

**FIGURE 1:**
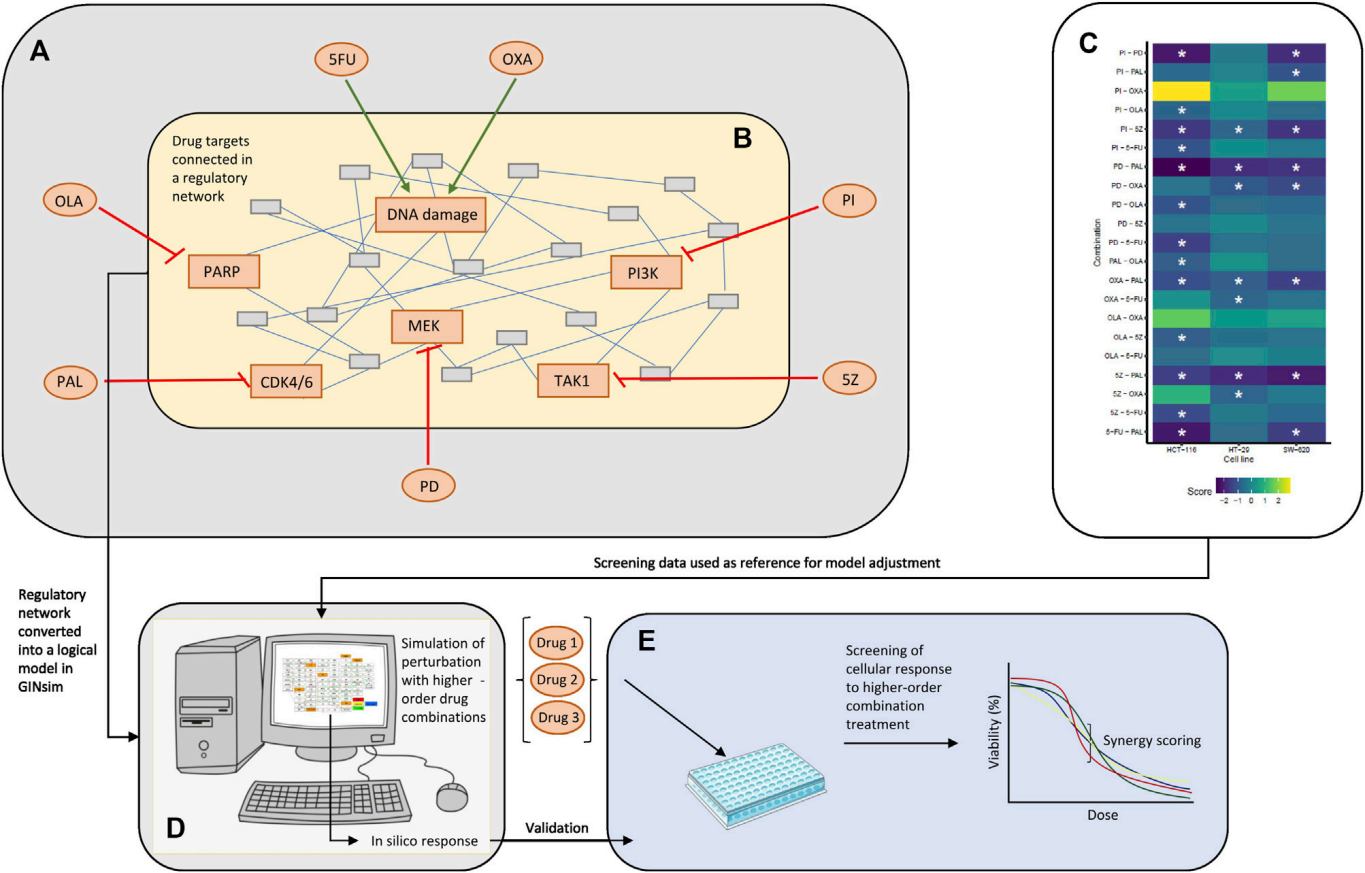
Overview of the study. **(A)** Drugs tested in single and combined (pairwise) in a high-throughput screen by Folkesson et al. (Folkesson et al., 2020). 5FU, OXA, PI, 5Z, PD, PAL, and OLA are abbreviations of 5-fluorouracil, oxaliplatin, PI-103, 5Z-7-oxozeaenol, PD-0325901, palbociclib and olaparib, respectively. **(B)** Regulatory network constructed around the targets of drugs tested in the published screen. The network in the figure represents an arbitrary network of nodes connected through undirected edges. **(C)** Combination effects observed in the screen, where asterisks mark combinations classified as synergistic (HSA score < −0.7) per cell line. Note that three cell lines (HCT-116, HT-29, and SW-620) were evaluated in the published combination screen, whereas the logical model was mainly adjusted for HCT-116 cells. **(D)** Logical model simulations of the regulatory network. The model was constructed in GINsim, adjusted based on response data from the screen **(C)** and used for prediction of response to higher-order drug combinations. **(E)** *In vitro* validation of cellular response to higher-order drug combinations tested *in silico* **(D)**.

A prior knowledge network was constructed based on information retrieved mainly from the databases KEGG (Kanehisa et al., 2017) and Signor (Perfetto et al., 2016), as well as from recent scientific literature. The network focused on the proteins/mechanisms and pathways targeted by drugs in our previously published high-throughput screen (Folkesson et al., 2020) (Figures 1A, C; Supplementary Table S1, Supplementary File S1: Results, figures, and tables). All interactions retrieved from public databases were reviewed, and for the initial model construction, the most well-described interactions were included. As three out of the seven drugs that were evaluated in our previously published screen are known to induce either DNA damage (5FU and oxaliplatin) or inhibition of DNA damage repair (olaparib), related pathways have been extensively described in our prior knowledge network, including any differences in induced damage, and activated repair mechanisms caused by the two DNA-damaging agents. See “Model adjustment” for more details. A complete list of references describing the molecular interactions included in this work can be found in Supplementary Table S2 (Supplementary File S1: Results, figures, and tables).

### Logical modelling

#### Construction of a logical model

In order to perform simulations of cellular behavior upon drug perturbations, the prior knowledge network was converted into a logical model in the software suite GINsim, version 3.0.0b (Gonzalez et al., 2006). In the logical model, components of the regulatory network (genes, proteins, complexes, chemical components/therapeutic agents, and phenotypes) are represented by nodes (connection points). Activating or inhibiting interactions are represented by edges. Apart from ‘Oxaliplatin’, ‘Fluorouracil’ and ‘Overall_phenotype’, all nodes are connected to a minimum of two other nodes *via* directed edges (a minimum of one incoming and one outgoing edge). ‘Oxaliplatin’ and ‘Fluorouracil’ act as input nodes, i.e., nodes fixed at a specified activity level, without incoming interactions and regulatory rules. ‘Overall_phenotype’ works as an output node, providing rapid access to the simulated results. The activity state (active/inactive) of non-input nodes is precisely defined by their regulatory nodes and a set of logical operators (AND (&), OR (|), NOT (!)), assembled into a logical rule that describes the conditions for activity of the target node. The overall model fate is determined by the output node ‘Overall_phenotype’, which can take on one of three levels (1, 2, 3). Apart from the output node, all nodes are Boolean. A complete summary of nodes and associated logical rules of the models is presented in Supplementary Table S3 (Supplementary File S1: Results, figures, and tables). The initial model contained 81 nodes and 142 edges.

#### Model adjustment

In the initial unadjusted model, the activity of all non-input nodes except DSB, representing double stranded DNA breaks, and Overall_phenotype followed a standard default (equation 1), Target node = (Activator 1 | Activator 2 | etc.) &! (Inhibitor 1 | Inhibitor 2 | etc.)! (1) i.e., for a node to be active, at least one of its activating regulators and none of its inhibiting regulators had to be active. We constructed the model so that the absence of perturbations, as well as perturbation of single nodes, resulted in an output corresponding to a proliferating cell phenotype (‘Overall_phenotype’ at its maximum level, 3). To meet the assumption of no overall effect of single node perturbations, subsequent model adjustments were guided by reference data (drug combination effects) generated from low to moderate drug concentrations, for which no significant effect on apoptosis was observed upon single-drug administration.

Following initial analysis, the model was adjusted in order to improve its ability to correctly classify synergistic and non-synergistic drug combinations observed in HCT-116 cells *in vitro* (Folkesson et al., 2020). The model was adjusted by stepwise implementation of one or several of the following changes.

- Changing of logical rule(s) for existing nodes.
- Addition of edge(s) between existing nodes.
- Addition of new nodes.

Each step was counted to keep track of the number of changes. Since the addition of a new node to a self-sustaining regulatory network involves addition of a minimum of two edges as well as modifications of at least one logical formula, we count these related changes together as one model adjustment. Likewise, since adding an edge between two existing nodes requires updating of the logical formula of the target node, these changes are counted as one model adjustment. In general, when adjusting a logical formula because of edge addition, the formula was first defined by the standardized form above (equation 1) (Mendoza and Xenarios, 2006). Any additional changes to the formula were counted as separate adjustments. Changes were made in the order in which they are listed in Supplementary Table S4 (Supplementary File S1: Results, figures, and tables). Overall, the order of model adjustments was motivated by the synergy scoring order of the combinations of interest (Supplementary Table S5, Supplementary File S1: Results, figures, and tables). The adjusted model consisted of 85 nodes and 158 edges. A complete summary of nodes and associated logical rules of the models (initial/adjusted) is presented in Supplementary Table S3 (Supplementary File S1: Results, figures, and tables). GINsim model definition files are available in the Figshare repository (see Data availability).

#### Stable state analysis and synergy quantification

An overall model output was defined based on upstream signaling component activities, and ranged from 1 to 3, where lower numbers correspond to growth-inhibited phenotypes and higher numbers correspond to growing phenotypes. To determine the level of the model output (1, 2, or 3), as well as the activity of non-input nodes at baseline and upon perturbation, stable states analysis was performed using the *Compute stable states* algorithm implemented in GINsim. By making use of so-called decision diagrams for representation of the Boolean functions of the model, the *Compute stable states* algorithm enables computation of stable state(s) from an initial given condition without the necessity to construct a state transition graph describing the dynamical behavior of the model (Naldi et al., 2007). This allows assessment of stable states also when working with larger networks where the construction and simulation of state transition graphs is not possible due to the size of the resulting graph. Due to the size of the network, all simulations were run using synchronous updating. In cases of multiple stable states, the *Assess attractor reachability* tool was used to estimate the probability of reaching each of the suggested stable states. Here, the evaluation of reachability is based on stochastic algorithms for evaluation of probabilistic outcome(s) resulting from repeated analysis of randomly sampled inputs (Naldi et al., 2018). Multiple such algorithms are implemented in GINsim and in this work, attractor reachability simulations were performed using the historically commonly featured *MonteCarlo algorithm*. The simulations were performed with 1,000 runs and a max depth of 1,000 as running parameters. Also here, the simulations were run using synchronous updating of the network. The stable state associated with the highest probability was selected as the main stable state. The level of the model output given by the stable state analysis was used for evaluation of combination effects *in silico*. As the baseline (unperturbed) model was associated with the highest output level (3) and since single-node perturbation did not have a reducing effect on the overall output level, perturbation of combinations of nodes which resulted in an output level ≤ 2 were, in the case of pairwise combinations, considered synergistic. Mathematically, this quantification of combination effects in simulations is analogous to synergy quantification using the Highest Single Agent (HSA) reference model, one of several mathematical reference models often employed for cell laboratory experiments (Lehár et al., 2007). Analyzed model perturbations and their *in vitro* counterparts (Folkesson et al., 2020) are listed in Supplementary Table S6 (Supplementary File S1: Results, figures, and tables). Nodes targeted by model perturbations adhere exclusively to the list of primary targets reported for each of the drugs that were tested *in vitro* (Supplementary Table S1; Supplementary File S1: Results, figures, and tables).

### Screening data

#### Reference data

##### Screening procedure

All details of the high-throughput screening procedure which provided response data used as reference for model adjustment can be found in the publication accompanying the dataset (Folkesson et al., 2020). Briefly, the authors studied the effect of seven single-drugs and all their pairwise combinations on the viability of three colorectal cancer (CRC) cell lines (HCT-116, HT-29, SW-620). The CellTiter-Glo 2.0 Viability Assay was used to assess viability of the cells following 48 h of drug treatment. In this article, we have mainly focused on data for HCT-116 cells.

##### Synergy scoring

The combination effects were quantified using the HSA reference model. HSA scores < −0.7 were considered synergistic, whereas everything else was considered non-synergistic. The rationale for the choice of the HSA model and identification of −0.7 as synergy cut-off value is given in *Quantification of synergy scores according to the HSA method* (Supplementary File S1: Results, figures, and tables).

### Validation data

#### Cell lines, drugs, and reagents

For *in vitro* validation of predictions made by the computational model, HCT-116 cells (CVCL_0291), obtained from the US National Cancer Institute (NCI), were used. Cells were cultivated in 1X RPMI-1640 medium (Thermo Fisher Scientific) supplemented with 10% fetal bovine serum (FBS, Sigma Aldrich), 2 mM L-Glutamine (Sigma Aldrich) and 100 U/mL Penicillin–Streptomycin (Thermo Fisher Scientific) and maintained at 37°C with 5% CO_2_ and 80% relative humidity. Passaging was performed according to in-house protocols as described in (Folkesson et al., 2020). Cells were used at passage numbers <20. Drugs used in the validation screen were olaparib (OLA), oxaliplatin (OXA), PI-103 (PI) (Selleckchem), PD0325901 (PD), 5-fluorouracil (5FU) (Sigma Aldrich) and 5Z-7-Oxozeaenol (5Z) (Enzo Life Sciences). The CellTiter-Glo 2.0 Viability Assay (Promega) was used for viability assessment of cells in the screen. A complete list of reagents and other material can be found in Supplementary Table SM1 (Supplementary File S2: Materials and Methods).

#### Screening procedure

For high-throughput validation screening of third-order combinations PI + PD + OXA, PI + PD+5FU, OLA + PI + PD, 5FU + OLA + PD, 5FU + OLA + PI and 5FU + OLA+5Z, HCT-116 cells were robotically seeded (Biomek FX^P^ Laboratory Automation Workstation; Beckman Coulter) with 35 µL complete growth medium in 384-well black tissue culture-treated plates (Corning) at a density of 1,200 cells/well. Following seeding, plates were shaken (1,600 rpm, 30s) to ensure uniform distribution of cells. The cells were then incubated for 24 h (37 °C with 5% CO2 and 80% relative humidity), whereafter drugs were added. Drugs (single, pairwise, and triple combinations), as well as positive (digitonin, staurosporine) and negative controls (DMSO) were added using a Tecan D300e system. The DMSO concentration in the culture medium never exceeded 0.5%. All treatment conditions are listed in Supplementary Table SM2 (Supplementary File S2: Materials and Methods). Details regarding dose selection can be found in *Selection of doses for third-order validation screening* (Supplementary File S1: Results, figures, and tables). Each condition (drug/combination, concentration) was tested in four technical replicates. Drug addition was followed by shaking of the plates (1,000 rpm, 30 s) and incubation at 37°C with 5% CO_2_ and 80% relative humidity. Following 48 h of treatment, cell viability was assessed using CellTiter-Glo 2.0, as described in (Folkesson et al., 2020). Cells were also imaged (2 sites per well) at 0, 24 and 48 h post drug addition. Imaging at 0 and 48 h was performed prior to drug addition and viability measurement, respectively. A SpectraMax i3x plate reader was used for all readouts. Two biological replicates of the high-throughput validation screen were performed.

For validation screening of third-order drug combinations OXA+5FU + PD and OXA+5FU + PI, and fourth-order combination OXA+5FU + PI + PD, HCT-116 cells were seeded with 90 µL complete growth medium in 96-well black tissue culture-treated plates (Corning) at a density of 4,800 cells/well. Seeding was followed by shaking of the plates (750 rpm, 30 s). The cells were then incubated for 24 h (37°C with 5% CO_2_ and 80% relative humidity), whereafter drugs (single, pairwise, triple and four-way combinations) and a negative control (DMSO) were added manually. All treatment conditions are listed in Supplementary Table SM3 (Supplementary File S2: Materials and Methods). Each condition (drug/combination, concentration) was tested in two technical replicates. Drug addition was followed by shaking of the plates (750 rpm, 60 s) and incubation at 37°C with 5% CO_2_ and 80% relative humidity. Following 48 h of treatment, cell viability was assessed using CellTiter-Glo 2.0. A POLARstar Omega plate reader was used for the readout. Three biological replicates were performed.

#### Data processing and statistical analysis

Confluency (high-throughput screen) was estimated by re-analyzing brightfield images using the SoftMax Pro 6.5.1 software. For each well, the percentage of covered area was estimated using settings specified in Supplementary Table SM4 (Supplementary File S2: Materials and Methods). For both confluency and viability (CellTiter-Glo 2.0), treatment effects were normalized to the plate-internal vehicle control (DMSO) and reported as average ± standard deviation. Pearson’s correlation coefficient has been used to quantify the association between variables (high-throughput screen) and biological replicates (high- and low-throughput screen).

#### Synergy scoring

The highest single agent reference model (Lehár et al., 2007) was used for evaluation of combination effects in the validation screens. HSA scores were computed by integrating the HSA excess value per data point to generate an overall HSA score for each combination across the tested doses. The latter was done to account for the fact that while effect size and synergy resulting from perturbations *in vitro* to some extent was dose-dependent, the corresponding perturbations *in silico* were logical to their nature. An integrated HSA excess score of 0 was used as cut-off, and combinations resulting in an integrated score < 0 and > 0 were deemed synergistic and antagonistic, respectively. Hence, in our *in vitro* experiments, synergy was called when the overall effect of the combination AB (for pairwise combinations), ABC (for triple combinations) and ABCD (for the four-way combination) was larger than the effect of the strongest underlying effector component (powerset), i.e., A and B for AB; A, B, C, AB, AC and BC for ABC; A, B, C, D, AB, AC, AD, BC, BD, CD, ABC, ABD, ACD, BCD for ABCD. Viability data (48 h) were used as the main data for assessment of combination effects. Confluency data were used for supplementary purposes, with combination effects evaluated at 24 h and 48 h. For each readout, combination effects were evaluated based on the average response of the biological replicates. In addition, for all tested higher-order combinations the statistical difference in relative viability between the highest-order combination *versus* the most effect underlying component (per dose) was calculated using a two-sided Student’s *t*-test. For each combination (third- or fourth-order) this was done per dose step and involved all technical replicates from all biological replicates.

#### Screening reproducibility

For the high-throughput screen, intra-experiment reproducibility was assessed for both viability and confluency data by computing the Pearson correlation between the two biological replicates. The correlation coefficients were 0.99 and 0.96 for viability and confluency, respectively (Supplementary Figure S4A, Supplementary File S1: Results, figures, and tables). Also, technical variability was assessed by first computing the coefficient of variation (CV) per treatment condition (4 replicates), followed by averaging of all CV values across biological replicate and readout (Supplementary Table S8 Supplementary File S1: Results, figures, and tables). The technical variability was considered low for both readouts with a CV of 6%–9%. In addition, inter-experiment reproducibility was assessed by comparing data points (viability data only) common for the reference screen (Folkesson et al., 2020) and the high-throughput validation screen. The correlation coefficient for responses tested in both setups was 0.96 (Supplementary Figure S4B; Supplementary File S1: Results, figures, and tables). Response assessed by viability was strongly correlated (0.96) with the confluency response (Supplementary Figure S5, Supplementary File S1: Results, figures, and tables), which was in concordance with results from the previous screen (Folkesson et al., 2020). For the low-throughput screen, intra-experiment reproducibility was assessed for viability data by computing the Pearson correlation between all pairs of biological replicates. The correlation coefficients ranged from 0.84 to 0.96 (Supplementary Figure S4C, Supplementary File S1: Results, figures, and tables).

## Results

### Overview of the study

The logical model presented in this paper was developed to computationally represent and analyze the joint influence of DNA damaging therapies and targeted therapies in pairwise, third- and fourth-order drug combinations. The model was built in the software suite GINsim (Gonzalez et al., 2006) and focused on the primary targets of drugs tested in our previously published combination screen (Folkesson et al., 2020) (Figures 1A, B). Following topological adjustments of the model, guided by drug response data of pairwise combination treatment of CRC cells (Figure 1C), we simulated response of HCT-116 cells to treatment with higher-order drug combinations (Figure 1D). Higher-order drug combinations identified as potential synergies by the model were further validated in an *in vitro* screen investigating the effect of higher-order drug combinations in HCT-116 cells (Figure 1E).

### Construction and initial analysis of the model

#### Description of the initial model

To adopt a regulatory network-based approach for the study of cellular effects of second and higher-order drug combinations we began with constructing a prior knowledge network encompassing all proteins and associated signaling pathways targeted by drugs in our previously published high-throughput combination screen (Folkesson et al., 2020) (Figures 1A, C). The resulting regulatory network comprised pathways mediating mitogenic signaling (MAPK, PI3K and TAK1/NF-kB pathways), pathways signaling the effect of DNA damage and repair induced by the chemotherapeutic agents oxaliplatin and 5FU (Bakkenist et al., 2018; Pilié et al., 2019), cell cycle components, and pathway-crosstalk. For more details on network construction, see *Details on network construction*, Supplementary File S1: Results, figures, and tables.

**FIGURE 2:**
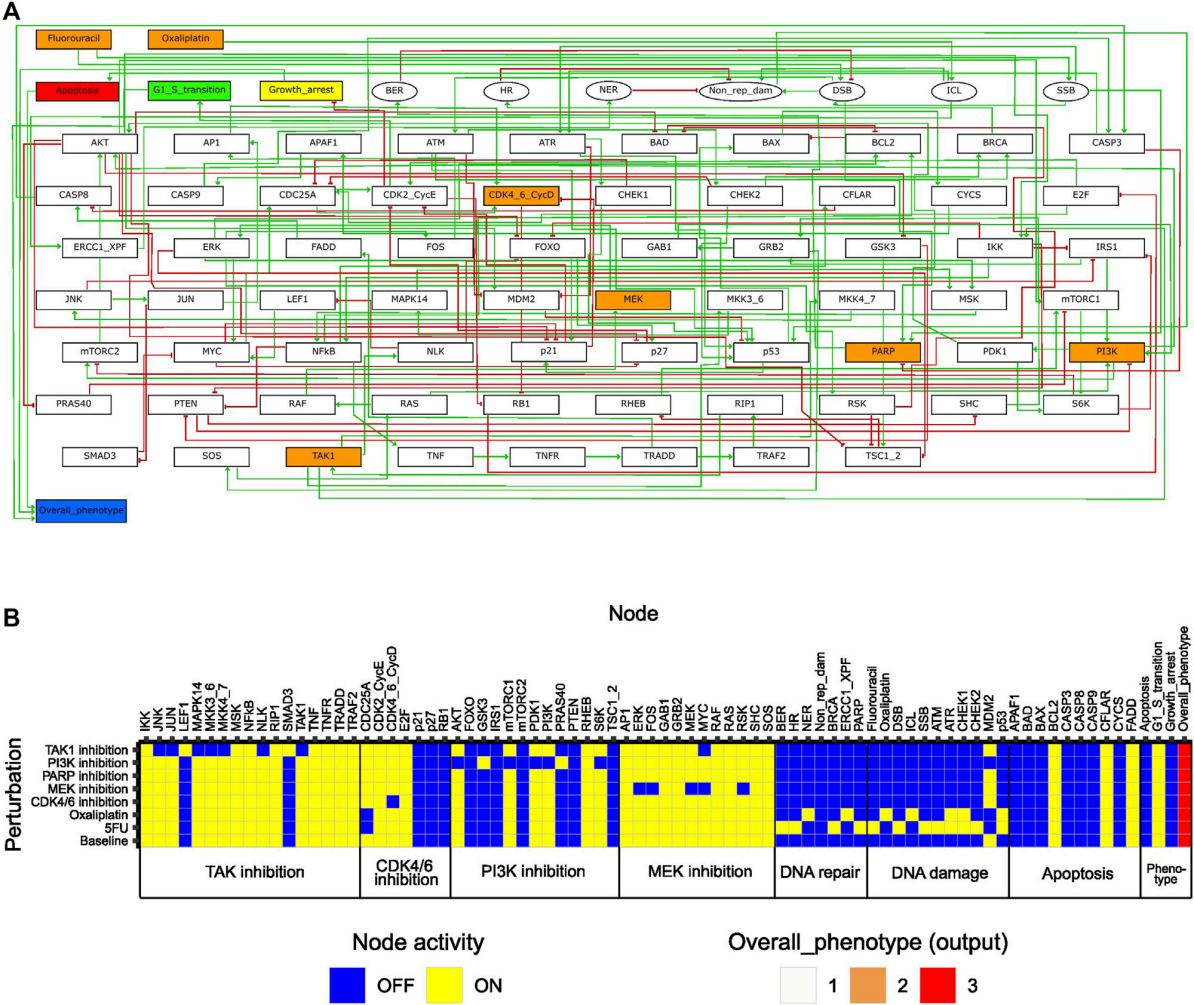
Features of the initial logical model. **(A)** Model topology; green arrows and red bar-headed lines indicate activation and inhibition, respectively. Orange rectangular nodes correspond to drugs (Fluorouracil/5FU, Oxaliplatin) or drug targets (MEK, PI3K, CDK4_6_CycD, TAK1, PARP; also signaling molecules). White rectangular nodes correspond to other signaling molecules. Elliptic nodes correspond to phenotypes or cellular events associated with DNA damage and repair. Nodes representing phenotypes apoptosis, growth arrest, G1/S transition and overall phenotype are colored in red, yellow, green, and blue, respectively. **(B)** Stable states (row-wise) computed with the initial model for all individual drug perturbations (single-drug effect), and for the unperturbed (baseline) model. Nodes are categorized based on their association with either a drug target or a phenotypic event.

To enable study of network behavior upon perturbations, we formulated the regulatory network as a logical model using GINsim (Figure 2A), as described in Materials and Methods. In the model, two input nodes represented the effect of the chemotherapeutic agents 5FU and oxaliplatin, whereas all other drug targets were represented by ‘internal’ nodes. For assessment of model phenotype at baseline and upon perturbation, three phenotypic pre-output nodes (representing G1/S transition, growth arrest, apoptosis) were included in the model. These nodes summarized into the output node ‘Overall_phenotype’, capable of taking on values 1, 2, and 3, where 3 corresponds to a proliferating cell. While a fourth output level, Overall_phenotype = 0, was defined as an output level resulting from inactivity of all pre-output nodes, this state was not achievable, since either “G1/S transition” or “Growth arrest” was defined to always be active.

#### Features of the initial model

To study the inherent signaling features of the initial model, prior to directed adjustments, model stable states were computed for several different conditions (Figure 2B). Here, ‘Baseline’ corresponds to what we would define as an unperturbed cell *in vitro* (i.e., no drugs added, and no ongoing DNA damage). The stable state associated with the ‘Baseline’ condition displayed activity of growth-promoting pathways and anti-apoptotic proteins, whereas pathways involved in DNA damage response and repair were all inactive (Figure 2B). The output value associated with the baseline stable state was the highest possible, which is expected as the features mentioned have been described for viable and growing cancer cells *in vitro* (Evan and Vousden, 2001; Fattahi et al., 2020; Guo et al., 2020; Mukhopadhyay and Lee, 2020). Using the initial computational model, we studied the *in silico* effects of all single drugs tested in our high-throughput screen (Folkesson et al., 2020). This was done by computing stable states in the presence of *in silico* perturbations that either inactivated nodes (MEK, PI3K, CDK4_6_CycD, TAK1, PARP) or fixed them in the active state (Fluorouracil, Oxaliplatin) (Supplementary Table S6, Supplementary File S1: Results, figures, and tables). Each such perturbation was associated with a single stable state (Figure 2B). A common observation with all perturbations was that although we observed an effect on the activity of components (nodes) within the pathway that was primarily targeted by each specific drug, the activity of nodes associated with other pathways was rarely affected. Additionally, none of the single drugs showed an inhibiting effect on the global model output (i.e., Overall_phenotype = 3). These aspects of our model are necessary prerequisites to enable detecting responses to combinatorial perturbations that exceed the response of single perturbations. Overall, the results from the analysis of the initial model indicated that the model correctly captured biologically plausible signaling features present in unperturbed cancer cells *in vitro*. Furthermore, upon single node inhibition or induction of DNA damage, the model accurately engaged response and repair mechanisms reported in the literature, still while allowing additional effects of combination treatment to be observed. Altogether, these results indicate that the model sufficiently represents the cell fate decision mechanism to study synergistic drug combinations affecting DNA integrity as well as cellular growth.

#### Model adjustment improves predictability

As we seek to use the computational model for prediction of cellular response to higher-order drug combinations, i.e., combinations comprising more than two drugs, we first improved the model’s ability to predict the response to pairwise drug combinations. This was done by implementation of adjustments in model topology and logical rules as described in Materials and Methods. Drug response data for HCT-116 cells from our previous combination screen (Folkesson et al., 2020) were used as reference data for model adjustments, and the effects of model adjustments on the output level were closely monitored by continually comparing stable state analysis to reference data. The reason for focusing on HCT-116 cells when performing model adjustment was that the strongest as well as the largest number of synergies were observed for this cell line in the reference screen. Despite this, we wanted to investigate if it was possible to detect an increased effect by the addition of a third and possibly fourth agent. More details concerning specific model adjustments are summarized in Supplementary Table S4 (Supplementary File S1: Results, figures, and tables).

**FIGURE 3:**
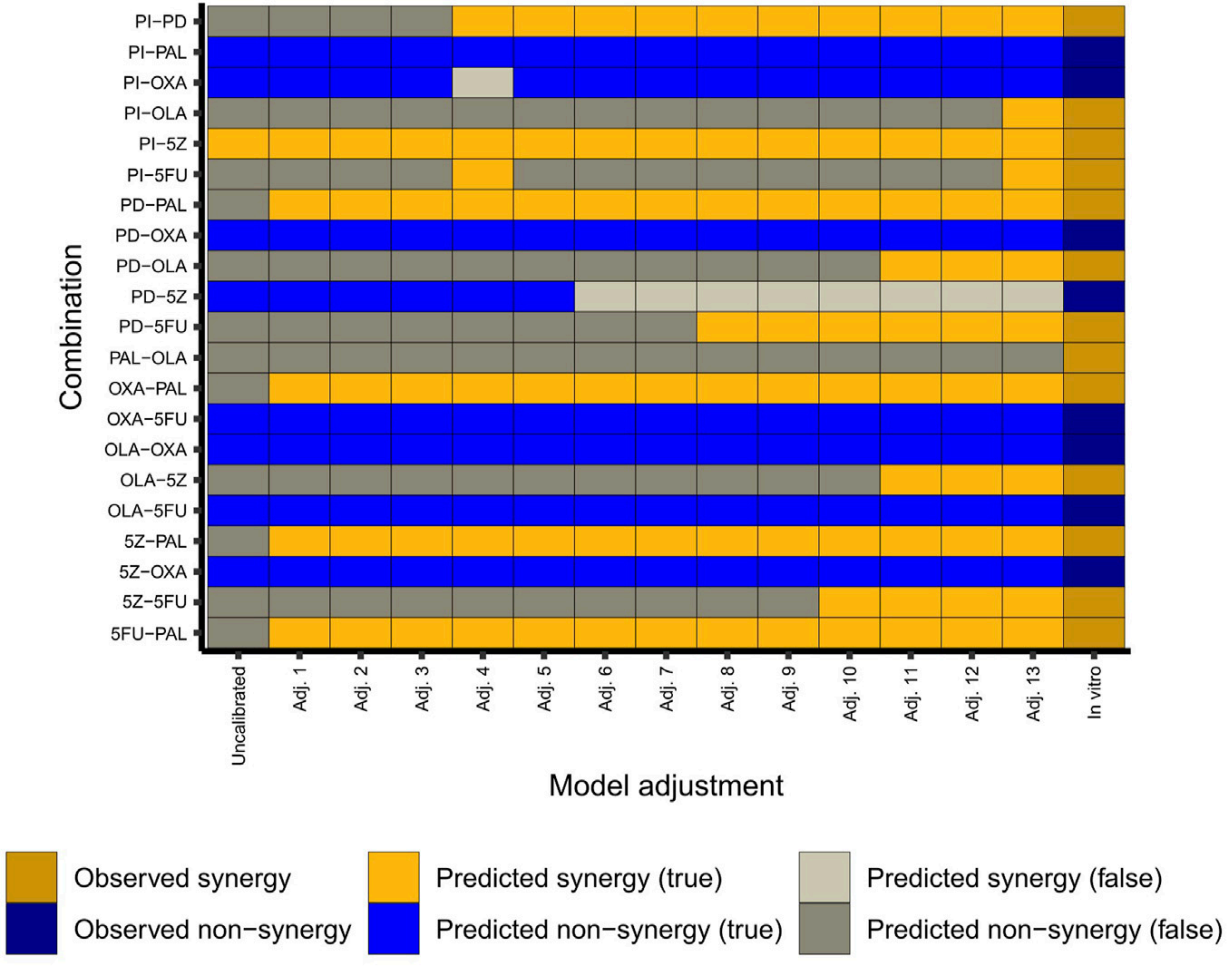
Adjustment of the model. The heatmap shows observed (*in vitro* in HCT-116 cells) and predicted (*in silico*) combination effects per adjustment step in the conversion of the original model into the final “HCT-116 adjusted” model. Adj. 13 corresponds to the adjusted (final) model. Details of the model adjustments are summarized in Supplementary Table S4 (Supplementary File S1: Results, figures, and tables).

We found that the model’s predictive capacity improved by each stepwise adjustment(s) guided by predictive performance for HCT-116 cells (Figure 3). Of note, the inclusion of the node MLH1 as a knockout (KO) played an important role for the predictive power of the adjusted model. Here, the node knockout represents the HCT-116 cells’ homozygous loss of MLH1 (Liu et al., 1995). Following implementation of this and other (in total 13) adjustments (Figure 3), the model correctly captured 12 out of 13 synergies, and 7 out of 8 non-synergies observed in HCT-116 cells *in vitro* (Folkesson et al., 2020). Of note, due to the inherent nature of the model construction, where single-node perturbations were set to have to have no effect on the overall phenotype, none of the antagonistic pairwise drug combinations detected in our previous screen (many of them involving the chemotherapeutic agent oxaliplatin) were predicted as antagonistic by the model. Instead, all pairwise perturbations which did not show an effect on overall phenotype were classified as “non-synergies”. However, as our primary aim was to use the model for prediction of synergistic higher-order combinations and since the model was now configured to closely mimic the synergy pattern of pairwise combinations in HCT-116 cells (Figure 3), we hypothesized that the model would also be effective for prediction of synergistic effects of higher-order drug combinations in the same cell line (HCT-116).

#### Pairwise combinations rarely induce apoptosis *in silico*

**FIGURE 4:**
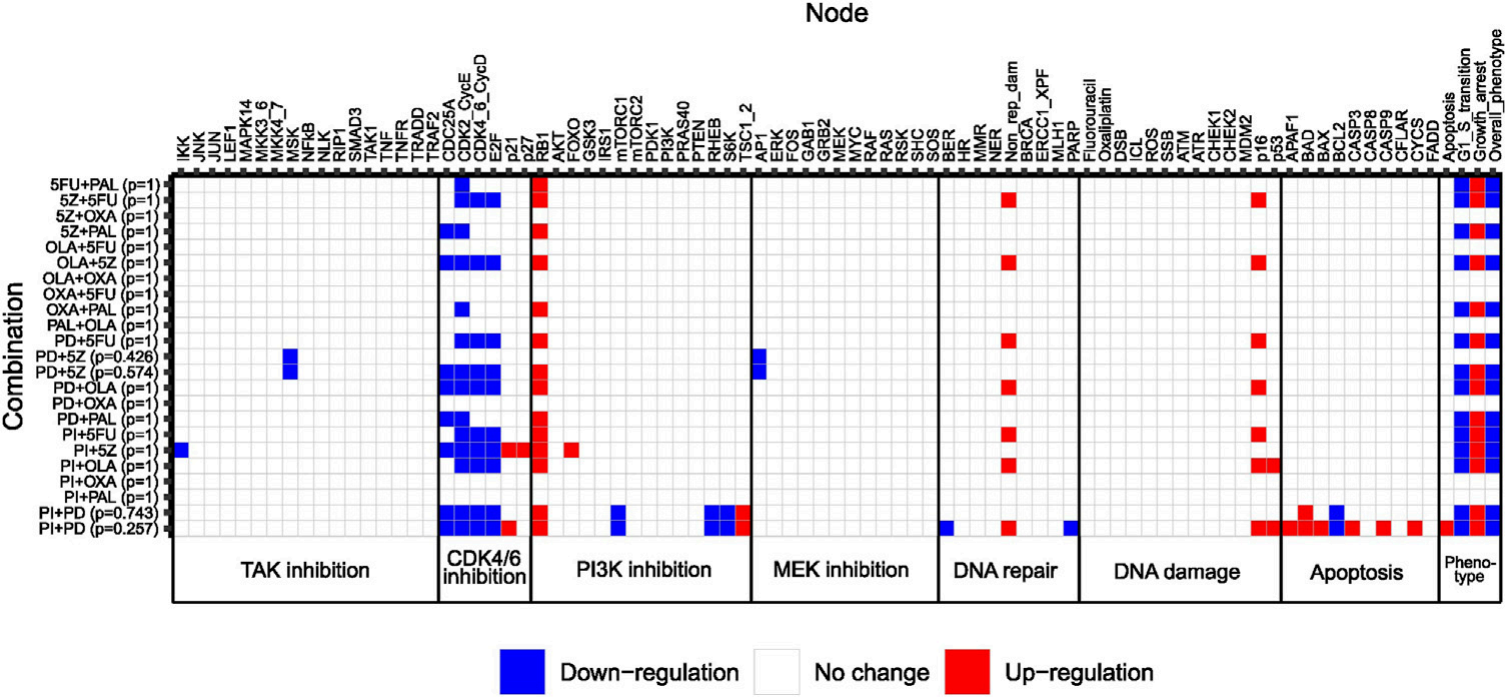
Heatmap showing differential activation of model nodes upon combined perturbation compared to single node perturbation in the adjusted model. Numbers between parentheses to the right of the combination name indicate the probability of reaching a specific stable state. Phenotypic output is shown to the right. Note that combinations classified as non-synergistic in the reference screen were also simulated for a confirmatory purpose.

Next, we used our adjusted computational model to study the effect size of pairwise combinations, to conclude whether synergistic higher-order combinations (>2 drugs) would at all be detectable by the model. To study the effect size of pairwise perturbations evaluated during model adjustment (Figure 3), associated stable states were computed for all combinations, followed by recording of the Overall_phenotype level and activities of model nodes. Maximum effect was defined as Overall_phenotype = 1, and hence an apoptotic phenotype, whereas Overall_phenotype = 2, characterized by cell cycle arrest in G1/S phase, was considered a medium effect. Perturbations with no effect on overall phenotype were associated with the highest possible value for Overall_phenotype, 3. Also, to gain further insights into possible mechanisms underlying observed synergistic effects of pairwise combinations, underlying single node perturbations were subjected to the same analysis (stable state analysis and recording of node activity/level), whereafter the differential activity of model nodes could be computed for all pairwise combinations as compared to the effect of underlying single node perturbations (Figure 4). We found that none of the 12 correctly predicted synergies entirely associated with the lowest output level that corresponds to an apoptotic phenotype, Overall_phenotype = 1 (Figure 4). Instead, we found that combined perturbations, when synergistic, often resulted in arrest of the cell cycle in G1/S phase (Figure 4), altogether resulting in the Overall_phenotype = 2. When studying model attractor reachability, one combination (PI + PD) was found capable of inducing apoptosis in a fraction of the simulations (Figure 4), but since the probability for such an outcome (assessed using the *Assess attractor reachability* algorithm in GINsim) was found to be less than 0.3, growth arrest (Overall_phenotype = 2) was selected as the main output also for this combination. The prediction of a cytostatic rather than cytotoxic synergistic effect of evaluated pairwise drug combinations was supported by data generated in the reference screen, which demonstrated an increase in confluency over time for all tested drug combinations (Supplementary Figure S6, Supplementary File S1: Results, figures, and tables). Here, a decrease in confluency relative to the starting point (drug addition) would have been sign of cytotoxicity. However, as no marker of apoptosis was included for most of the combinations evaluated in the reference screen, an additional apoptotic effect *in vitro* cannot be ruled out. Computationally, as none of the pairwise perturbations tested by the model did on their own induce a maximum effect (apoptosis/Overall_phenotype = 1), such an effect induced by e.g., perturbation of additional nodes would theoretically still be detectable. We therefore consider the model in its current adjusted state to be well suited for exploring combination effects of higher-order drug combinations in HCT-116 cells.

The model was next used for prediction of synergistic third and fourth-order combinations in HCT-116 cells. Combinations subjected to stable state analysis and all associated stable states are listed in Supplementary Table S9 and Supplementary Table S10 (Supplementary File S1: Results, figures, and tables). For third and fourth-order combinations, synergy was called when the effect of the highest-order combination was larger than the effect of the most effective lower-order treatment subset.

**FIGURE 5:**
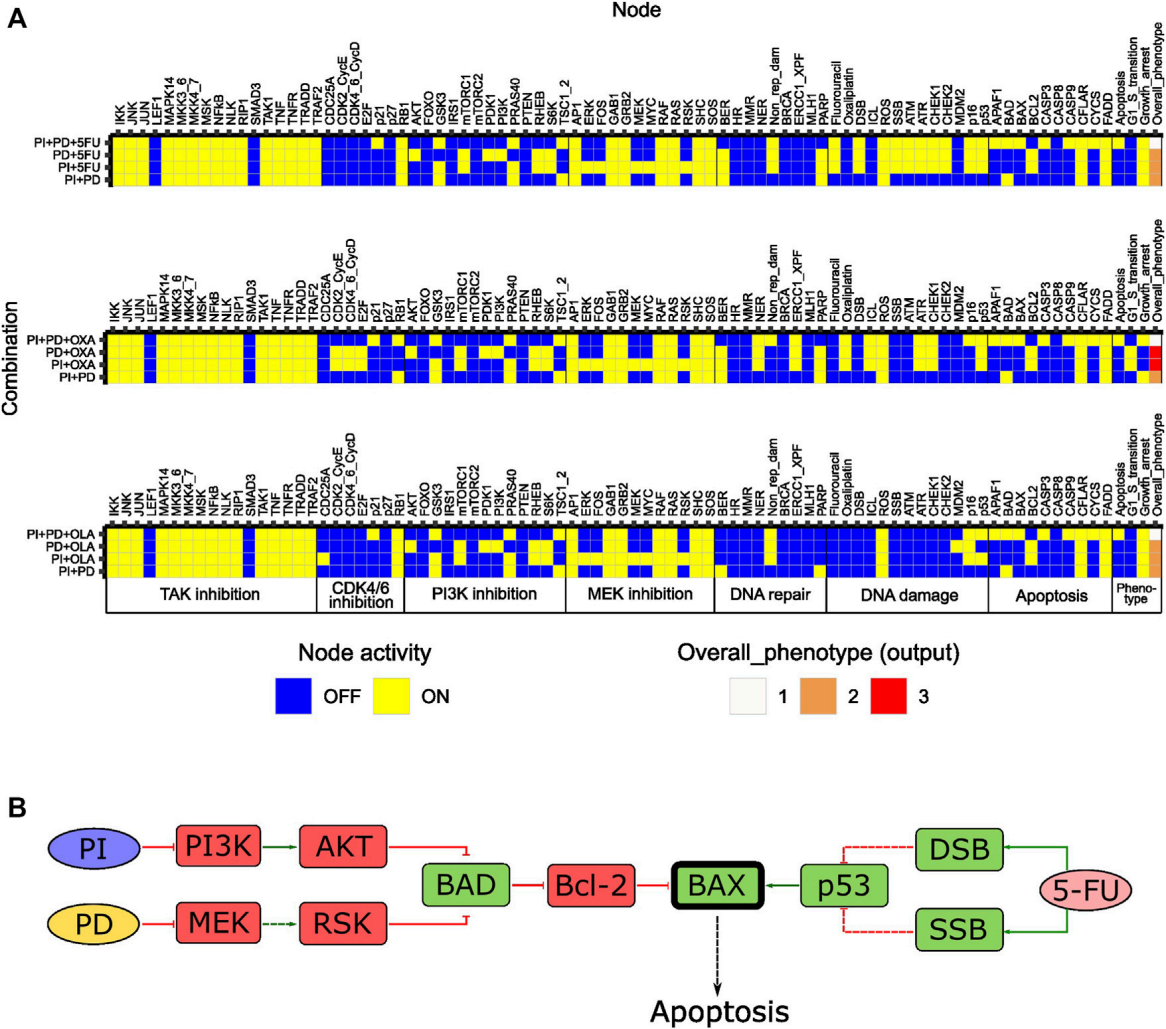
In silico effects of third-order drug combinations predicted for HCT-116 cells. **(A)** Stable states (row-wise) computed with the adjusted model for synergistic third-order drug combinations and associated second-order combinations. The probability for the stable state of the third-order drug combination was 1 in all three cases. **(B)** Suggested mechanism accounting for the synergistic effects of third-order combinations in **(A)**, exemplified for PI + PD+5FU. Synergistically activated BAX is highlighted in bold. Dashed arrows/bar-headed lines indicate indirect links between nodes.

From 35 possible third-order drug combinations tested, three (PI + PD+5FU, PI + PD + OXA and PI + PD + OLA) were predicted as synergistic by the model (Figure 5A, Supplementary Table S9, Supplementary File S1: Results, figures, and tables). In all three cases, the probability of the stable state for the third-order drug combination was 1. Of note, all three synergistic third-order combinations involved combined inhibition of PI3K and MEK (PI + PD). Two of them also involved chemotherapeutic DNA damage. All three combinations were associated with Overall_phenotype = 1, and an apoptotic phenotype. According to the model, apoptosis was enabled by the activity of the pro-apoptotic factor BAX, which was synergistically activated upon third-order perturbation, but not by any of the simulated single or pairwise drug responses (Figure 5B). The model suggested that the presence of functional BAX was crucial for synergy of these third-order combinations. The latter was confirmed by performing simulations where the activity of BAX was turned off (knocked out), whereupon none of the three combinations showed synergy (results not shown). The suggested mechanism is supported by literature stating that higher-order drug combinations might be needed in order to effectively kill CRC cells and that direct targeting of processes such as apoptosis and cell cycle might be crucial for enhanced cytotoxicity (Horn et al., 2016). According to further stable state analysis of all possible fourth-order perturbations (35), none of these combinations contributed with any additional effect compared to third or second order combinations (Supplementary Table S10, Supplementary File S1: Results, figures, and tables).

Altogether, results from the tests indicate that the adjusted model can be used for prediction of especially third-order drug combinations with possible synergistic effects in HCT-116 cells.

#### Screening reveals synergistic effect of third-order drug combinations in HCT-116 cells *in vitro*

**FIGURE 6:**
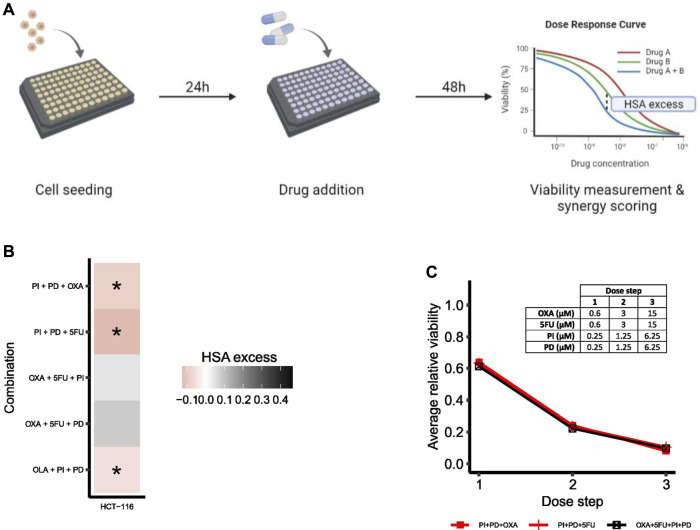
Drug synergy validations. **(A)** Simplified overview of the screening procedure with synergy scoring exemplified for a second-order combination. **(B)** Heatmap of HSA excess (viability-based) summed across the dose-ray per combination. Asterisks (*) indicate combinations predicted to be synergistic by the computational model. **(C)** Viability dose-response curve for HCT-116 cells treated with the fourth-order combination OXA+5FU + PI + PD. The plot shows the fourth-order combination and the two most potent lower-order combinations (PI + PD + OXA, PI + PD+5FU). Concentrations per dose step are presented in the table. To make the plot clearer, error bars are not included.

To evaluate the predictive performance of the adjusted model, the effect of higher-order drug combinations in HCT-116 cells was assessed by a follow-up validation screen. An overview of the general screening procedure is shown in Figure 6A. During screening, cells were treated with all third-order drug combinations predicted to be synergistic by the model (PI + PD + OXA, PI + PD+5FU, OLA + PI + PD), two randomly selected third-order combinations with predicted non-synergy (OXA+5FU + PD, OXA+5FU + PI), one of the predicted fourth-order non-synergies (OXA+5FU + PI + PD), as well as all underlying lower-order treatments. Combination effects of second- and higher-order drug combinations were evaluated based on viability data by calculating the HSA excess per dose step (see illustration of the meaning of HSA excess in Figure 6A), followed by integration of HSA excess values across the entire dose range per combination, as described in Materials and Methods. We applied a cut-off at HSA = 0 for classification of synergy for higher-order drug combinations; hence, drug combinations with integrated HSA excess scores < 0 and > 0 were considered synergistic and antagonistic, respectively. While HSA as a reference model for synergy scoring often is considered less stringent in its classification of synergy compared to several other reference models (such as the Bliss independence and Loewe additivity models), and therefore should be used with extra care, its mathematical analogy to how we in this study defined synergy *in silico* made it suitable for quantification of synergy also *in vitro*. A complete list of all treatment conditions evaluated in the validation screen can be found in Supplementary Table SM2 and Supplementary Table SM3 (Supplementary File S2: Materials and Methods).

All three third-order combinations predicted as synergies by the computational model were confirmed statistically significant synergistic (*p* < 0.05) at multiple doses in HCT-116 cells *in vitro* in the high-throughput validation screen (Figure 6B; Supplementary Figure S7 and Supplementary Table S11, Supplementary File S1: Results, figures, and tables). We also found that the two combinations OXA+5FU + PI and OXA+5FU + PD, which were both predicted as non-synergies by the model, were non-synergistic in HCT-116 cells *in vitro* (Figure 6B; Supplementary Figure S8 and Supplementary Table S12, Supplementary File S1: Results, figures, and tables), indicating that the adjusted model has the power to correctly predict synergistic, as well as non-synergistic properties of multiple third-order combinations in HCT-116 cells.

To hypothesize on further model refinements related to modelling of DNA repair, we also screened three additional third-order drug combinations; 5FU + OLA + PD, 5FU + OLA + PI and 5FU + OLA+5Z. The computational model predicted none of these combinations to be synergistic, as 5FU combined with either of PD, PI and 5Z alone resulted in non-repairable DNA damage (Figure 4). Hence, the model did not indicate additional repair-compromising effects of PARP inhibition (OLA), although this could be expected from a biological point of view (see *Selection of additional third-order drug combinations for screening*, Supplementary File S1: Results, figures, and tables). These combinations were found to be synergistic when screening HCT-116 cells (Supplementary Figure S7), in line with biological reasoning, and indeed indicating that certain predictions most likely would benefit from a multileveled representation of non-repairable DNA damage.

Based on model predictions, none of the potential fourth-order combinations (35) were expected to act synergistically in HCT-116 cells (Supplementary Table S10, Supplementary File S1: Results, figures, and tables) and due to the experimental complexity of fourth-order combinations (14 effector components to be individually tested per fourth-order component), we chose to limit the experimental follow-up to only one such combination, OXA+5FU + PI + PD. The selected fourth-order combination displayed a negligible negative HSA excess value in HCT-116 cells (Figure 6C).

To summarize, our combined computational-experimental framework predicted and validated three novel third-order drug combinations demonstrating synergistic effects in HCT-116 cells.

## Discussion

Computational models hold great promise as supportive tools within many fields of medicine. Models presented in the medical literature span a wide range of application areas, from studies of disease development (Baratchart et al., 2015) to biomarker discovery (Ding et al., 2019) and treatment prediction (Eduati et al., 2020; Folkesson et al., 2020; Kuenzi, 2020; Niederdorfer et al., 2020; Tsirvouli et al., 2020). Prediction of treatment response is of special importance within the field of cancer, where the time spent on ineffective therapy will have severe consequences as it may allow disease progression beyond treatment. Targeting cancer using combinations of drugs has proven to be an efficient strategy to improve therapy response (Yu et al., 2008; Zhang et al., 2009; Halilovic et al., 2010; Haagensen et al., 2012; Holt et al., 2012; Flanigan et al., 2013), but unfortunately, this method poses a huge challenge to laboratory technicians and decision-making doctors in clinical settings, as the number of available drug targets and combinations is practically without limits. Here, predictive computational models offer a useful alternative as they can efficiently rationalize drug screening (Flobak et al., 2015; Eduati et al., 2020; Niederdorfer et al., 2020).

Multiple models have successfully been used for studies of the effect and mechanisms of single and pairwise drug combinations (Flobak et al., 2015; Niederdorfer et al., 2020), but few and to our knowledge exclusively statistical models have been optimized for studies of higher-order drug combinations. In this study, we have therefore focused on developing a computational mechanism-based model aimed to predict the effect of higher-order drug combinations in CRC. The topology of our model encompasses signal transduction pathways frequently dysregulated in cancer (MAPK/ERK, PI3K/AKT and TAK1/NF-kB), as well as pathways underlying DNA compromising and repair effects of two chemotherapeutic agents (5FU, oxaliplatin). We believe that our model provides an important improvement over existing models, where the majority have focused on modelling either traditional signal transduction (Grieco et al., 2013; Flobak et al., 2015; Niederdorfer et al., 2020) or DNA damage (Mombach et al., 2014; Mombach et al., 2015; Gupta et al., 2020c; Gupta et al., 2020b; Gupta et al., 2020a), but rarely both.

Aiming to ultimately use our model to predict effects of higher-order drug combinations, we used data from a previously published screen of pairwise combinations (Folkesson et al., 2020) as guidance when adjusting the model topology. In the present study, the topology was updated with the intention to correctly classify the combination effects (synergy/non-synergy) of as many as possible of the pairwise combinations tested in HCT-116 cells in the screen. Subsequently, and without further adjustments, the model was used to predict effects of third- and fourth-order combinations. We found that, in general, topology adjustments required for prediction of high-scoring pairwise synergies (Supplementary Table S5, Supplementary File S1: Results, figures, and tables) could easily be supported by the literature, whereas adjustments introduced to predict lower-scoring synergies were considerably more exploratory. An example of the latter was the inclusion of senescence-related components, together with BRCA-activating interactions from MEK, MYC and PI3K, to make the model predictive of the three pairwise synergies PD+5FU, 5Z+5FU and PI+5FU. In the model, absence (inactivation) of any of MEK, MYC and PI3K leads to irreparable DNA damage in the presence of 5FU, thereby suggesting that these components might represent mechanisms for the synergistic effects of PD+5FU, 5Z+5FU and PI+5FU.

Our approach of manually reconstructing the model topology to fit the model behavior to data is to be regarded as effect-based, rather than mechanistic, as no or very little molecular data (mutational status etc.) of unperturbed cells were used. The adjustments implemented in the refined version of the model were the ones that we considered had the largest effect on the predictive power of the model. Here, the inclusion of the node MLH1 as knockout (KO) was found to play an important role for the predictive power of the model, whereas the ectopic expression of PIK3CA, another well-known mutation of HCT-116 cells, was not - hence the decision was made not to include the latter as a model adjustment. In other words, no traditional model calibration, like the one described by Niederdorfer et al. (2020) and Flobak et al. (2015), was performed. Instead, the refinement strategy presented here is dependent on the availability of two-way dose-response data. While this might reduce some of the model’s applicability in cases where no such data are available, the result is in line with what has previously been published by others, where mathematical models’ ability to predict the effect of higher-order drug combinations has been found to be dependent on calibration using a minimum of two-way dose-response data (Geva-Zatorsky et al., 2010; Zimmer et al., 2016; Zimmer et al., 2017; Katzir et al., 2019).

By adjusting the model to fit data from HCT-116 cells in our previous screen of pairwise combinations (Folkesson et al., 2020), we were able to predict three synergistic third-order combinations from a total of 35 combinations. In this context, it is worth mentioning that since the screening data that were used as reference for the model adjustment were generated based on pairwise perturbations using relatively few drugs (seven), and all of them with unique primary targets, it might be that the performance of the adjusted computational model to some extent also reflects the potency of the exact drugs that were used to generate the reference data. For example, the in the reference screen the tested MEK1/2 inhibitor PD0325901 (PD) was found to on its own have a significant impact on viability. This might explain the absence of an additional synergistic effect for several of the combinations where this drug was involved in the drug screen. It could be speculated that the use of a less potent MEK inhibitor, such as e.g., cobimetinib, would have resulted in other synergistic drug combinations, which in turn would have affected the model adjustment strategy and possibly also the model’s ability to predict the effect of higher-order drug combinations.

No fourth-order combinations (out of 35 tested) were predicted to be synergistic. While the identification of synergistic fourth-order combinations was not possible for combinations where underlying combinations (pairwise and third-order) did on their own induce maximum effect (apoptosis), this would still have been technically possible for fourth-order combinations where none of the underlying combinations induced an effect larger than G1 arrest. No such synergistic fourth-order were however predicted. Overall, the predicted frequency of synergistic higher-order combinations was unexpectedly low, compared to results from e.g., a study by Tekin et al. (2018), where the frequency of higher-order synergies was found to increase with the number of drugs. The small number of predicted synergies might have several explanations. First of all, the topology of our model was constructed mainly around the targets of drugs tested in the previous screen (Folkesson et al., 2020), meaning that several pathways with known relevance for CRC (e.g., Wnt and Notch signaling pathways) were not included. It is possible that if we, already when constructing the first topology, would have included components more distantly related to drug targets, we also would have been able to capture effects currently not manifested in the model. The same goes for model edges. The edges connecting nodes in the initial unadjusted model were edges which are all frequently described in the literature in relation to the modelled pathways. Less well-described edges were not included at the initial stage but were considered, however not necessarily included, during model adjustment. Likely is also that the inclusion of additional output phenotypes, such as growth arrest in G2/M phase, as well as an allowance for the model to take on multiple simultaneous phenotypes upon perturbation (e.g., apoptosis and G1/S transition), would have contributed to larger search space for any synergies to be found. Future iterations of the model should therefore focus also on the simulation of G2 arrest. In addition, for more precise simulation of specific molecular mechanisms/phenotypes, such as apoptosis, future modelling attempts should make use of calibration data from screens where these mechanisms have been evaluated on a molecular level in addition to currently used readout methods.

Although the limited amount of higher-order response data from validation screening prevented us from performing a full evaluation of the model’s predictive performance for HCT-116 cells, the obtained results support the predictive capacity of the model: all three predicted third-order synergies were confirmed *in vitro* in HCT-116 cells. We also found that three of the predicted non-synergies demonstrated non-synergistic effects *in vitro*. The clinical relevance of the model is manifested in the three correctly predicted third-order synergies, which all encompass at least one clinically approved drug. In this context, it should however be mentioned that none of the by *in vitro* tests observed synergies (HSA < 0) demonstrated statistically significant synergistic effects over the whole tested dose range. This highlights that while computational modelling may increase the efficiency of *in vitro* screening, detecting synergies by screening is still not a trivial undertaking.

While we in this study focused exclusively on the possible therapeutic benefits provided by the use of higher-order drug combinations, we also need to be aware of the possibility of different toxicity profiles for higher-order drug combinations, that could potentially cause severe side-effects when used clinically. Due to the choice of only engaging documented primary targets when performing *in silico* perturbations, *in vitro* effects resulting from off-target engagement were likely not correctly depicted. We expect that prediction also of side-effects of treatment will call for the use of computational methods. Altogether, we believe that our model and our modelling strategy holds great promise as an *in silico* pre-selection tool when searching the vast combinatorial space for synergistic higher-order combinations, as well as for further investigations of molecular mechanisms underlying higher-order synergy.

## Conclusion

Results from our study demonstrate that our model well represents the response behavior of HCT-116 cells and has the capability to in the same cell line correctly predict synergistic effects, and possibly also non-synergistic effects of third-order drug combinations. Altogether, the model may serve as a solid base for further studies of mechanistic effects of third and higher-order drug combinations.

## Data Availability

All data supporting the conclusion of the article are available in the Figshare repository (https://figshare.com/s/2795463950dfe7e1e804).
