# Supplementary material for: Synergistic effects of complex drug combinations in colorectal cancer cells predicted by logical modelling
Source: Front Syst Biol. 2023 Feb 27;3:1112831. doi: 10.3389/fsysb.2023.1112831 (PMC12342003; doi:10.3389/fsysb.2023.1112831)
Supplement: Supplementary file 1 [file Image1.pdf]

Supplementary Material

Synergistic effects of complex drug combinations in colorectal cancer cells predicted by logical modelling

Evelina Folkesson, B. Cristoffer Sakshaug, Andrea D. Hoel, Geir Klinkenberg, Åsmund Flobak*[*]

*** Correspondence:** Åsmund Flobak, asmund.flobak@ntnu.no

1 Supplementary file 1: Results, figures, and tables

1.1 Supplementary results

1.1.1 Quantification of synergy scores according to the HSA method

In the publication by Folkesson et al. (Folkesson et al., 2020), the combination effect of drugs was evaluated according to the Bliss independence model. The computational model presented in our study classifies combination effects based on an approach that is more analogous to that of the Highest Single Agent (HSA) model, and therefore we chose to re-quantify the combination effects of the previously published screening data using the HSA model. HSA scores were computed condition-wise by 1) calculating the HSA excess value per data point (i.e., dose combination) in the original matrix, and 2) summing the HSA excess values per matrix to generate a matrix HSA score. The matrix HSA score was then used for further classification of combination effects. We identified the most synergistic combinations by classifying combinations with a matrix HSA score ≤ -0.7 as synergistic and everything else as non-synergistic. The cut-off value -0.7 was selected by evaluating accuracy, as well as the difference between true (TPR) and false positive rates (FPR) for cut-off values ranging from ≤ -2.6 (all combinations classified as non-synergies) to ≤ 2.9 (all combinations classified as synergies). Bliss classification labels from the screen (Folkesson et al., 2020) were used as "real" values. Using a matrix HSA score of -0.7 as cut-off resulted in the highest accuracy (Figure S1A), as well as the largest difference between true and false positive rates (Figure S1B). In general, for cut-off values tested here, the discriminating power of the HSA reference model was found to be higher than that of a random classifier (AUC = 0.88; Figure S1C). Using a matrix HSA score of -0.7 as cut-off, 28 synergistic conditions were identified (Figure S1D). Note that in the screen by Folkesson et al. three cell lines were tested: HCT-116, HT-29, and SW-620. Reference synergy data for all three cell lines are shown in Figure S1A, even though we have primarily focused on data from HCT-116 cells in this study.

1.1.2 Selection of doses for third-order validation screening

Doses selected for third-order combination screening (validation screening) were based on a ray design (Foucquier and Guedj, 2015). Selected doses were guided by the effect of included lower-order components (single drugs and pairwise combinations) when tested in HCT-116 cells in the screen by Folkesson et al. (Folkesson et al., 2020). For each of the third-order combinations, the included components (i.e., the third-order combination, the three pairwise combinations and the three single-drugs) were screened at four dose steps as presented in Table M2 (Supplementary file 2: Materials and Methods). For single-drugs, selected concentrations were the same as tested in the screen by Folkesson et al. The dose steps for the pairwise combinations corresponded to the 'diagonal' of the corresponding full matrix in the Folkesson screen.

Before executing the third order combination screen, we verified that selected concentrations would not result in larger effects of single drugs and pairwise combinations than what would allow for an additional effect of the third-order combination to be detected (Figure S2). We also observed that pairwise combinations classified as synergies based on the full-matrix design were, compared to full-matrix non-synergies, associated with considerably lower HSA excess (stronger synergy) also across the ray (Figure S3). Also, when ranking pairwise combinations based on ray- and full-matrix synergy score (HSA excess), these placed relatively equal (Table S7, Supplementary file 1: Results, figures, and tables). Note that all analysis related to selection of doses was performed using the data from the screen by Folkesson et al. (Folkesson et al., 2020).

1.1.3 Details on network construction

3 out of 7 single drugs included in the screen (Table S1, Supplementary file 1: Results, figures, and tables) were aimed at either inducing DNA damage (5FU, oxaliplatin) or inhibiting DNA damage repair (olaparib/PARP inhibitor). Therefore, we sought to construct associated pathways as detailed and representative as possible, taking agent-specific damage and repair mechanisms into account. We found that oxaliplatin reportedly induces inter- and intra-strand crosslinks (ICL) (Bakkenist et al., 2018), whereas treatment of cells with 5FU is associated with single (SSB) and double strand breaks (DSB) (Bakkenist et al., 2018). Furthermore, we found that distinct mechanisms were described for the repair of DNA damage induced by 5FU and oxaliplatin, respectively: nucleotide excision repair (NER) was reported to be responsible for repairing ICLs induced by oxaliplatin, whereas base excision repair (BER; for SSBs) and homologous recombination (HR; for DSBs) were most frequently described for the repair of 5FU-induced damage (Bakkenist et al., 2018). The distinct damage and repair pathways described for the two chemotherapeutic agents were all included in the network, as were components with documented significance for the function of the repair pathways (PARP, BRCA, and ERCC1/XPF for BER, HR and NER, respectively). Also, our network incorporated a feature where SSBs were allowed to be converted into DSBs in the absence of BER; DSBs which are then repaired by HR (Rose et al., 2020).

1.1.4 Selection of additional third-order drug combinations for screening

As relatively few third-order and none of the tested fourth-order combinations were found to be synergistic according to our computational model, we wished to hypothesize whether any of the third-order combinations, which were *not* predicted as synergistic by the model, could still hold promise based on mechanisms or information currently not expressed by our model. Here we paid extra attention to the three combinations 5FU+PD+OLA, 5FU+PI+OLA and 5FU+5Z+OLA. The reason for this focus was the suggested activating effect of MEK, MYC and PI3K on BRCA (Chen et al., 2011; Ibrahim et al., 2012; Rehman et al., 2012; Vena et al., 2018), as well as 5FU's well-known ability to induce DNA damage in the shape of both DSBs and SSBs (Bakkenist et al., 2018). We hypothesized that upon treatment of cells with 5FU, some cells would contract DSBs, others SSBs, and yet others both DSBs and SSBs. In the presence of active MEK, MYC, PI3K and PARP, and hence functional HR and BER pathways, both types of DNA damage would be repairable (Figure S9, i.). In the case of non-functional BER, SSBs would be converted into DSBs (Rose et al., 2020). As HR would at this point however still be functional, DSBs would be repairable (Figure S9, ii.). This would explain why the combination 5FU+OLA did not manifest any synergistic effects in the screen by Folkesson et al. (Folkesson et al., 2020). In the same screen, all of PD+5FU, PI+5FU and 5Z+5FU were however classified as synergistic, indicating that PD, PI and 5Z or any of their downstream targets possibly had an inhibitory effect on the repair of DSBs induced by 5FU. As BER was on the other hand expected to be functional for all these combinations, cells only affected by SSBs would not succumb to treatment with these pairwise combinations (Figure S9, iii.). We hypothesized that a total effect of 5FU treatment would instead be reached if 5FU treatment took place in the absence of *both* HR and BER pathways (Figure S9, iv). As the adjusted model suggested that MEK, MYC and PI3K were all essential for the activity of HR, whereas PARP was needed for BER, third-order combinations involving 5FU, olaparib and any of MEK, MYC and PI3K (i.e., 5FU+PD+OLA, 5FU+PI+OLA and 5FU+5Z+OLA) would therefore possibly display synergistic effects, although the dynamics of our model did not allow such effects to be detected.

1.2 Supplementary figures

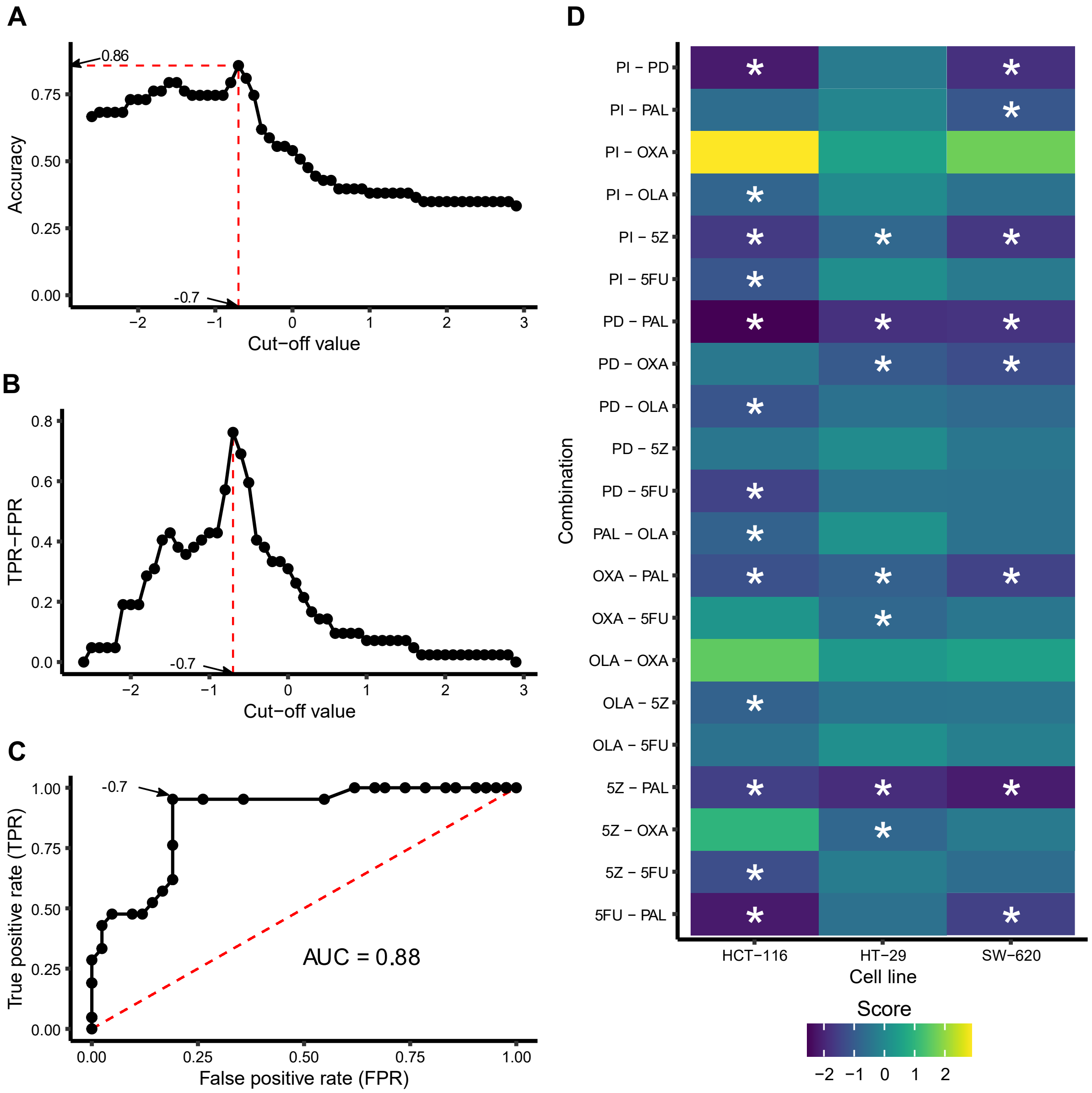

Figure S1. Identification of cut-off value for classification of synergies using the HSA reference model. **(A)** Plot showing Accuracy vs. Cut-off value. The cut-off value resulting in the highest accuracy (HSA excess = -0.7) is indicated on the x axis and with a red dashed line. The corresponding accuracy (0.86) is indicated on the y axis and with a red dashed line. **(B)** Plot showing TPR-FPR vs. Cut-off value. The cut-off value that maximizes TPR-FPR (HSA excess = -0.7) is indicated on the x axis and with a red dashed line. **(C)** Receiver operating characteristic (ROC) curve for prediction of combination effect using the HSA reference model. The data point generated by the cut-off value -0.7 is indicated (arrow). The red dashed line represents a random classifier. **(D)** Heatmap presenting cell line-wise HSA excess scores for the 21 drug combinations tested in the screen. Asterisks indicate combinations classified as synergies when -0.7 is used as the cut-off value (synergy for HSA excess ≤ -0.7).

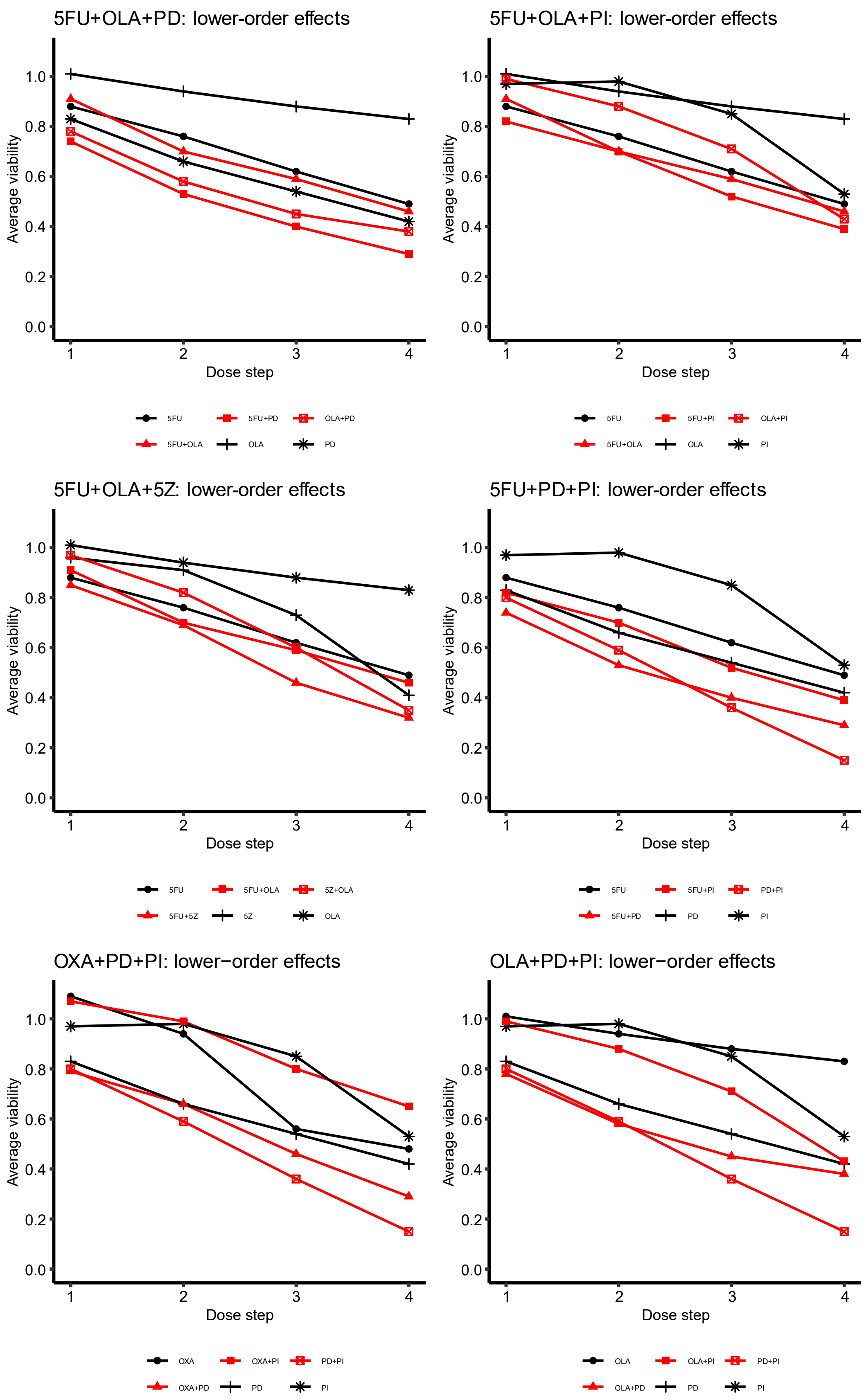

Figure S2. See next page.

Figure S2. Dose-response curves for single-drugs (black) and pairwise combinations (red) associated with the in titles given third order combinations (selected for testing in the high-throughput validation screen). Dose steps 1-4 correspond to drug-specific concentrations presented in Table M2 (Supplementary file 2: Materials and Methods). Curves shown here are based on HCT-116 cell line data from the screen by Folkesson et al. (Folkesson et al., 2020).

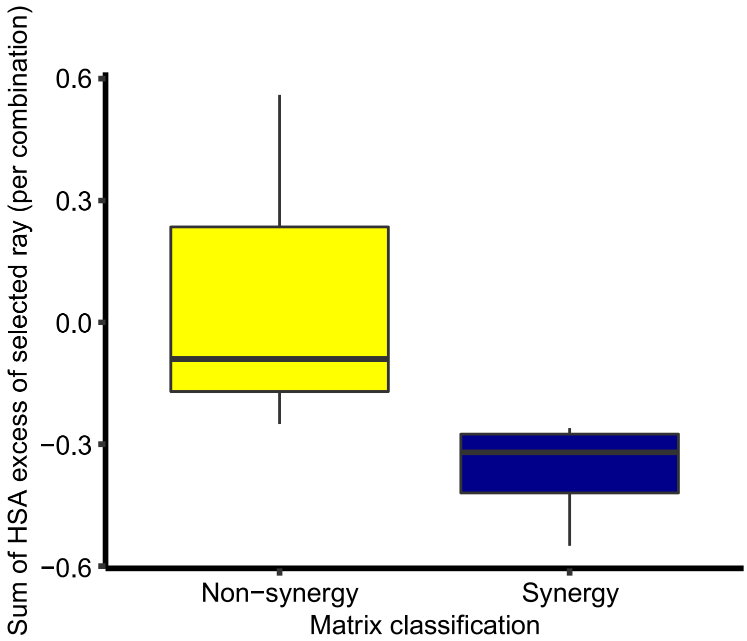

Figure S3. Summed ray HSA excess (HCT-116 cells) for pairwise drug combinations listed in Table M2 (Supplementary file 2: Materials and Methods). Combinations are boxed (non-synergy/synergy) according to full-matrix synergy classification. The analysis was performed using the data from the screen by Folkesson et al. (Folkesson et al., 2020).

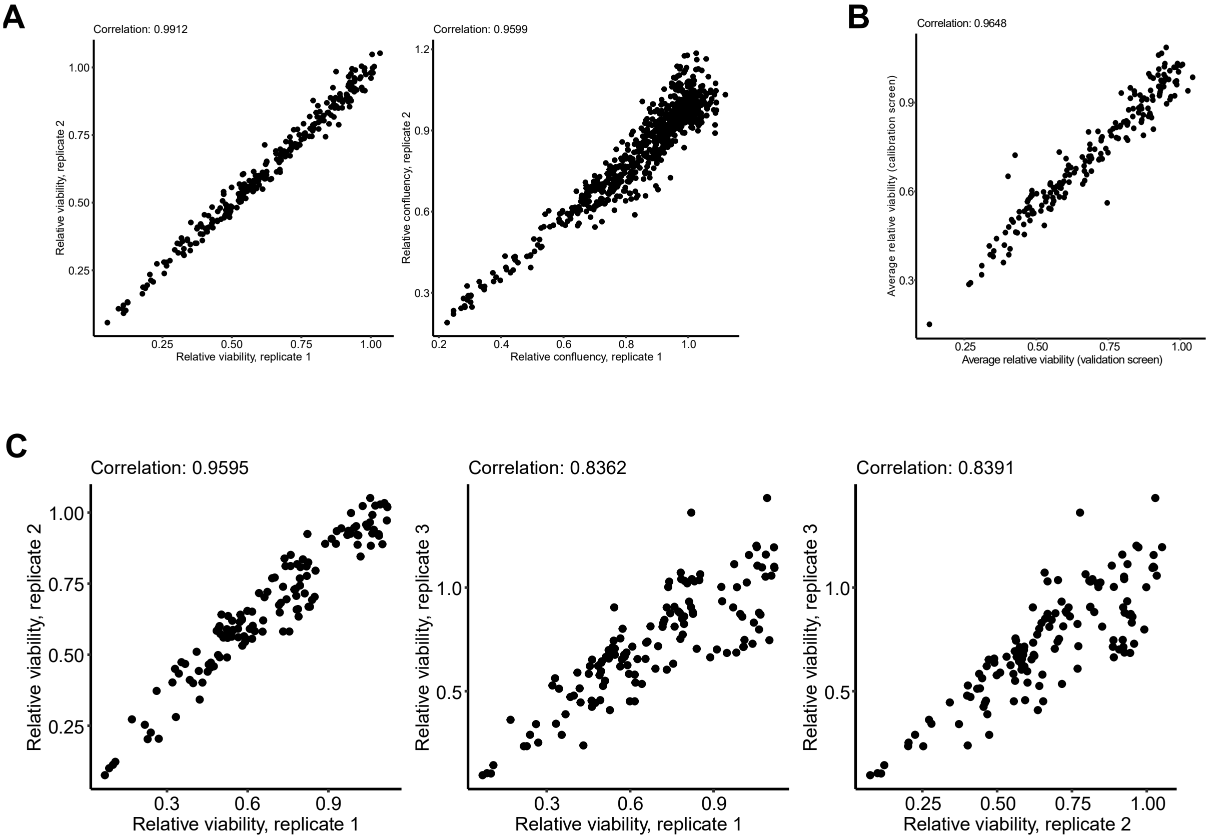

Figure S4. Intra and inter-experiment reproducibility visualized in correlation plots. **(A)** Correlation between biological replicates (intra-experiment reproducibility) in the high-throughput validation screen (left = viability, right = confluency). **(B)** Correlation between average relative viability in the high-throughput validation screen and average relative viability in the reference/calibration screen (inter-experiment reproducibility). **(C)** Correlation between biological replicates (intra-experiment reproducibility) in the low-throughput validation screen (viability data).

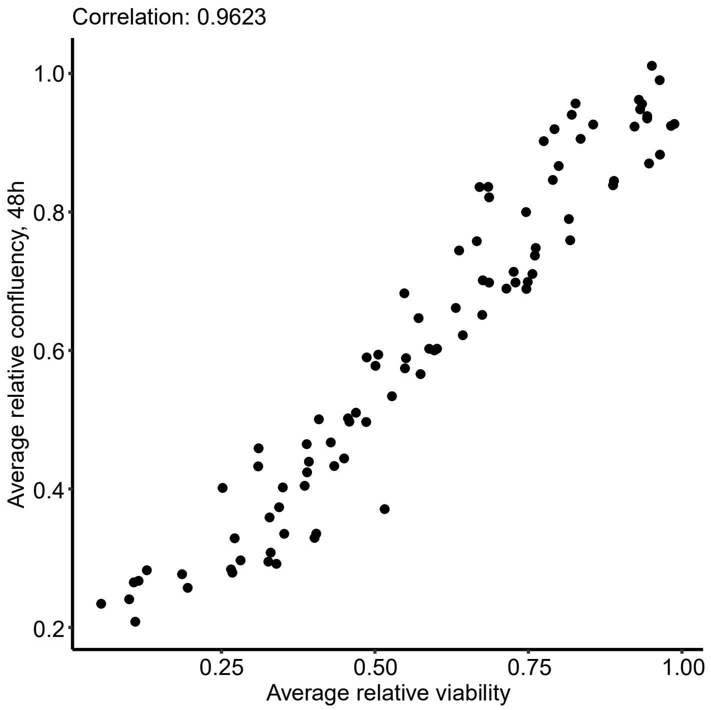

Figure S5. Response correlation obtained with different readouts (viability, confluency) at 48 hours post drug addition in the high-throughput validation screen.

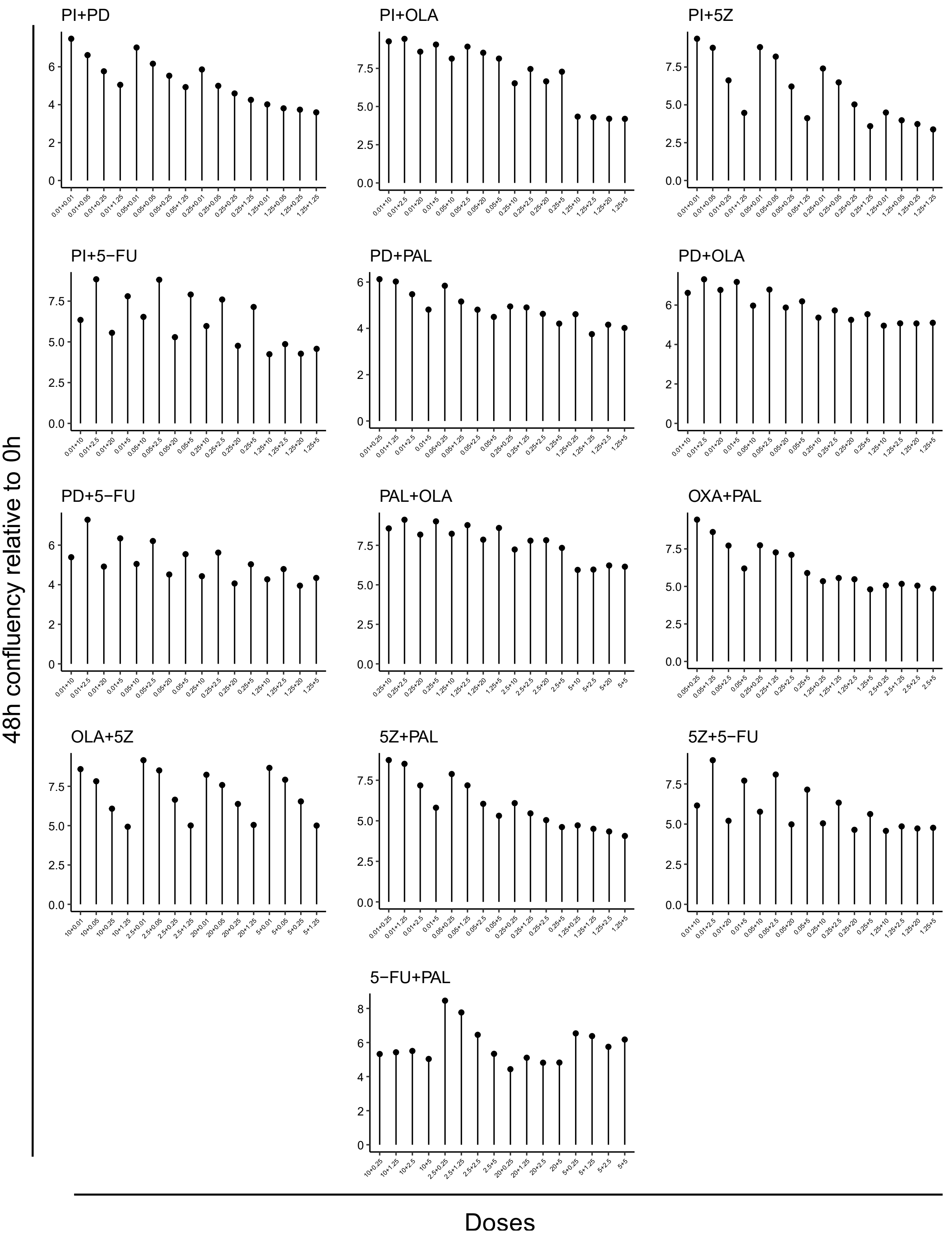

Figure S6. Lollipop plots showing the confluency at 48h relative to 0h (drug addition) for each tested dose (dose combination with doses in µM) per synergistic combination in the reference screen. Doses are specified per drug in the same order as these are listed in the plot titles, i.e., 10+0.25 for the combination 5-FU+PAL corresponds to a combination of 10 µM 5-FU + 0.25 µM PAL. Plots were generated based on data from the screen by Folkesson et al. (Folkesson et al., 2020).

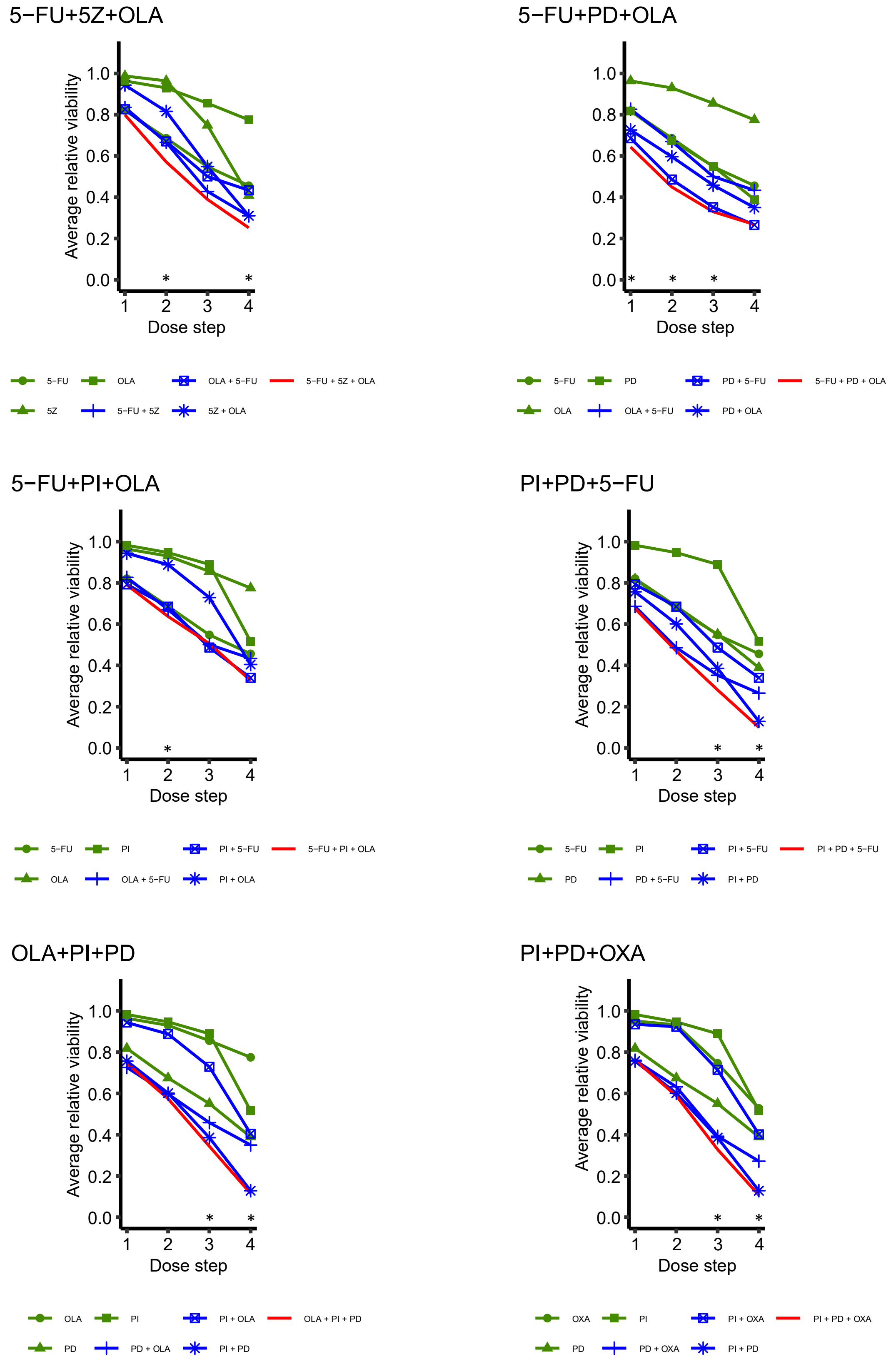

Figure S7. Viability dose-response curves for treatment with third-order combinations and underlying lower-order combinations/single drugs in HCT-116 cells in the high-throughput validation screen. To make plots clearer, error bars are not included. Asterisks indicate a significantly ($p<0.05$) stronger synergistic effect of the third-order combination compared to the strongest underlying component. Statistics are calculated dose-wise across all technical replicates from two biological replicates. For more information about statistical testing, see Materials and Methods and Table S11.

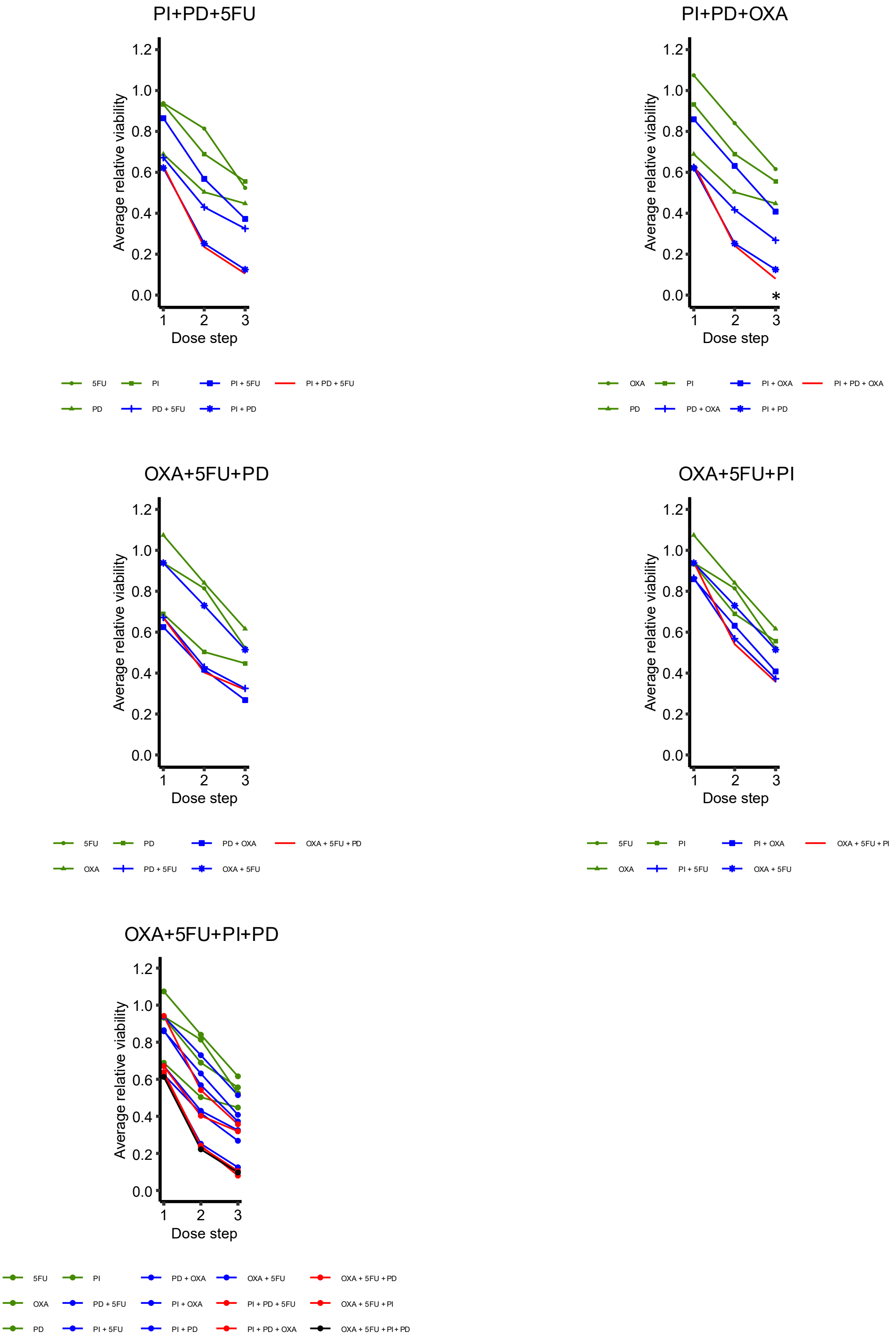

Figure S8. Viability dose-response curves for treatment with third and fourth order combinations and underlying lower-order combinations/single drugs in HCT-116 cells in the low-throughput validation screen. To make plots clearer, error bars are not included. Asterisks indicate a significantly ($p<0.05$) stronger synergistic effect of the highest-order combination compared to the strongest underlying component. Statistics are calculated dose-wise across all technical replicates from three biological replicates. For more information about statistical testing, see Materials and Methods and Table S12.

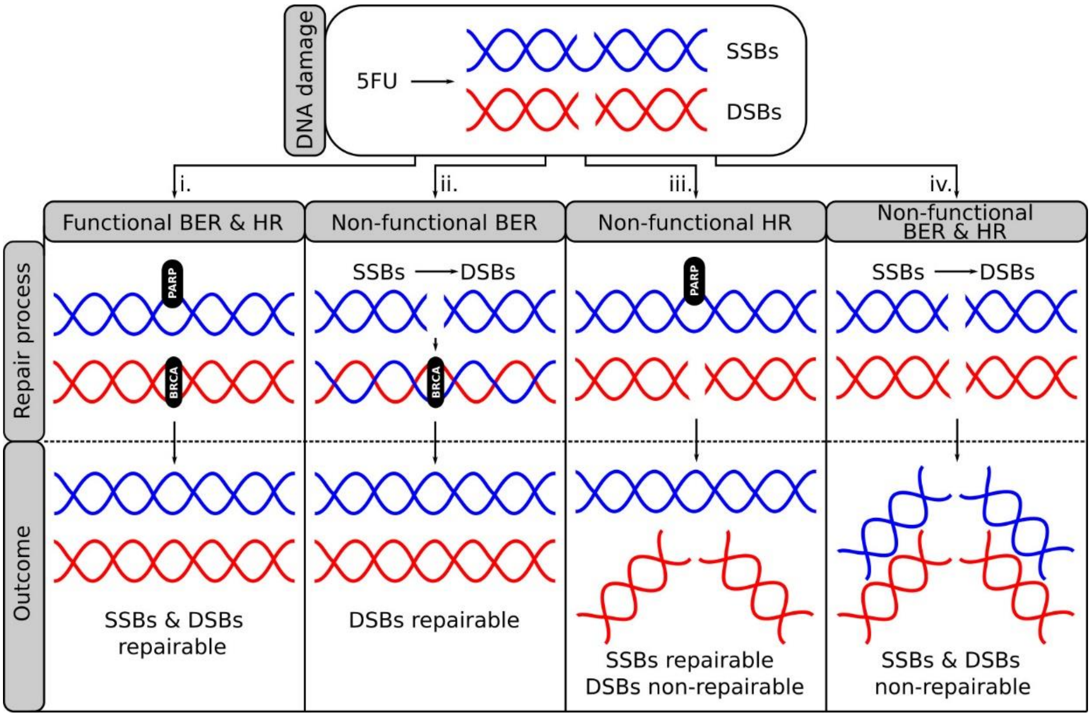

Figure S9. Hypothesized effects of 5FU treatment in the presence and absence of functional DNA damage repair pathways (BER, HR).

1.3 Supplementary tables

Table S1. List of drugs included in the high-throughput screen by Folkesson et al. (Folkesson et al., 2020), their primary targets (according to manufacturer), and nodes targeted in the model (targeting strategy; KO = knockout, E1 = ectopic activity). Abbreviated drug names are given in brackets in the *Drug name* column.

**Drug name**	**Primary target**	**Node targeted in the model**
5-fluorouracil (5FU)	Thymidylate synthase (DNA replication and transcription)	Fluorouracil (E1)
Olaparib (OLA)	PARP1/2	PARP (KO)
Oxaliplatin (OXA)	DNA replication and transcription	Oxaliplatin (E1)
Palbociclib (PAL)	CDK4/6	CDK4_6_CycD (KO)
PD0325901 (PD)	MEK	MEK (KO)
PI-103 (PI)	PIK3CA/PIK3CB/PIK3CD/PIK3CG	PI3K (KO)
5Z-7-oxozeaenol (5Z)	TAK1	TAK1 (KO)

Table S2. List of references (PubMed ID and/or database/source) associated with nodes and interactions contained by any of the models in this work. 1 = initial model, 2 = adjusted model.

**Node**	**Effector node(s)**	**Regulation**	**PubMed ID**	**Database/source**	**Model**
AKT	mTORC2	Positive; phosphorylation	15718470	Signor, KEGG, PubMed	1, 2
	PDK1	Positive; phosphorylation	12808134	Signor, KEGG, PubMed	1, 2
	PI3K	Positive; phosphorylation	12167717	Signor, KEGG, PubMed	1, 2
AP1	FOS	Positive; binding	25875593	Signor, PubMed	1, 2
	JUN	Positive; binding	25875593	Signor, PubMed	1, 2
APAF1	CYCS	Positive; binding	15829969	Signor, KEGG, PubMed	1, 2
Apoptosis	CASP3	Positive; upregulation	14585074	Signor, KEGG, PubMed	1, 2
ATM	DSB	Positive; sensing/detection of DNA damage	24003211	PubMed	1, 2
ATR	ICL	Positive; sensing/detection of DNA damage	24003211	PubMed	1, 2
	SSB	Positive; sensing/detection of DNA damage	24003211	PubMed	1, 2

BAD	AKT	Negative; phosphorylation	9346240	Signor, KEGG, PubMed	1, 2
	RSK	Negative; phosphorylation	10837486	Signor, KEGG, PubMed	1, 2
BAX	BCL2	Negative; binding	8183370	Signor, KEGG, PubMed	1, 2
	p53	Positive; binding	16151013	Signor, KEGG, PubMed	1, 2
BCL2	BAD	Negative; re-localization	15694340	Signor, KEGG, PubMed	1, 2
	ERK	Positive; phosphorylation	10669763	Signor, PubMed	1, 2
	NFkB	Positive; transcriptional regulation	11704864	KEGG, PubMed	1, 2
BER	PARP	Positive; binding	28412778	KEGG, PubMed	1, 2
BRCA	ATM	Positive; phosphorylation	10550055	Signor, PubMed	1, 2
	CHEK2	Positive; phosphorylation	14701743	Signor, PubMed	1, 2
	MEK	Positive; unknown	29545922	PubMed	2
	MYC	Positive; unknown	21668996	PubMed	2
	PI3K	Positive; unknown	22915752, 23148373	PubMed	2

	ROS	Positive; unknown	24704793	PubMed	2
CASP3	CASP8	Positive; cleavage	16964285	Signor, KEGG, PubMed	1, 2
	CASP9	Positive; cleavage	15657060	Signor, KEGG, PubMed	1, 2
CASP8	CFLAR	Negative; binding	14585074	Signor, KEGG, PubMed	1, 2
	FADD	Positive; binding	11717445	Signor, KEGG, PubMed	1, 2
CASP9	APAF1	Positive; binding	15829969	Signor, KEGG, PubMed	1, 2
CDC25A	CHEK1	Negative; phosphorylation	20068082	Signor, KEGG, PubMed	1, 2
	CHEK2	Negative; phosphorylation	12676583	Signor, KEGG, PubMed	1, 2
	E2F	Positive; transcriptional regulation	11154267	Signor, PubMed	1, 2
	MYC	Positive; transcriptional regulation	11154267	Signor, PubMed	1, 2
CDK2_CycE	E2F	Positive; transcriptional regulation	8649818	Signor, KEGG, PubMed	1, 2
	CDC25A	Positive; dephosphorylation	12411508	Signor, KEGG, PubMed	1, 2

	p16	Negative; binding	10022885	Signor, PubMed	2
	p21	Negative; binding	16982699	Signor, KEGG, PubMed	1, 2
	p27	Negative; binding	17409098	Signor, KEGG, PubMed	1, 2
CDK4_6_CycD	AP1	Positive; transcriptional regulation	12509763	Signor, PubMed	1, 2
	CDC25A	Positive; dephosphorylation	23429262	Signor, KEGG, PubMed	1, 2
	mTORC1	Positive; translational regulation	17724476	Pubmed	2
	p16	Negative; binding	11154267, 8891723, 10022885	KEGG, PubMed	2
	p21	Negative; binding	7626805	Signor, KEGG, PubMed	1, 2
CFLAR	NFkB	Positive; transcriptional regulation	11463813	KEGG, PubMed	1, 2
CHEK1	ATR	Positive; phosphorylation	15775976	Signor, KEGG, PubMed	1, 2
CHEK2	ATM	Positive; phosphorylation	10973490	Signor, KEGG, PubMed	1, 2
CYCS	BAX	Positive; re-localization	10629050	Signor, KEGG, PubMed	1, 2

DSB	BER	Negative; prevents further damage	33015058	PubMed	1, 2
	Fluorouracil	Positive; induces damage	30316683	PubMed	1, 2
	SSB	Positive; conversion in the absence of repair	33015058	PubMed	1, 2
E2F	CDK4_6_CycD	Positive; activation	7970707	PubMed	2
	RB1	Negative; binding	8255752	Signor, KEGG, PubMed	1, 2
ERCC1_XPF	ICL	Positive; activation	30316683, 20682979	PubMed	1, 2
ERK	MEK	Positive; phosphorylation	11730323	Signor, KEGG, PubMed	1, 2
FADD	TRADD	Positive; binding	8565075	Signor, KEGG, PubMed	1, 2
Fluorouracil	-	-	-	-	1, 2
FOS	ERK	Positive; phosphorylation	12972619	Signor, KEGG, PubMed	1, 2
FOXO	AKT	Negative; phosphorylation	19188143	Signor, KEGG, PubMed	1, 2
	IKK	Negative; phosphorylation	19188143	Signor, KEGG, PubMed	1, 2
	NLK	Negative; phosphorylation	-	KEGG	1, 2

G1_S_transition	CDK2_CycE	Positive; upregulation	21524151	Signor, KEGG, PubMed	1, 2
GAB1	GRB2	Positive; binding	12766170	Signor, KEGG, PubMed	1, 2
GRB2	SHC	Positive; binding	17306385	Signor, KEGG, PubMed	1, 2
	IRS1	Positive; activation	-	KEGG	1, 2
Growth_arrest	CDK2_CycE	Negative; downregulation	21524151	Signor, KEGG, PubMed	1, 2
GSK3	AKT	Negative; phosphorylation	23552696	Signor, KEGG, PubMed	1, 2
HR	BRCA	Positive; recognition and execution	12947386	KEGG, PubMed	1, 2
ICL	Oxaliplatin	Positive; induces damage	30316683	PubMed	1, 2
IKK	AKT	Positive; phosphorylation	19609947	Signor, KEGG, PubMed	1, 2
	TAK1	Positive; phosphorylation	11460167, 16953224	Signor, KEGG, PubMed	1, 2
IRS1	IKK	Negative; phosphorylation	-	KEGG	1, 2
	JNK	Negative; phosphorylation	-	KEGG	1, 2

	S6K	Negative; phosphorylation	18952604	KEGG, PubMed	1, 2
JNK	MKK4_7	Positive; phosphorylation	11062067	Signor, KEGG, PubMed	1, 2
JUN	JNK	Positive; phosphorylation	17158707	Signor, KEGG, PubMed	1, 2
LEF1	NLK	Negative; phosphorylation	12556497	Signor, KEGG, PubMed	1, 2
MAPK14	MKK3_6	Positive; phosphorylation	8622669	Signor, KEGG, PubMed	1, 2
MDM2	AKT	Positive; phosphorylation	15169778	Signor, KEGG, PubMed	1, 2
	ATM	Negative; phosphorylation	19816404	Signor, PubMed	1, 2
	ATR	Negative; phosphorylation	14654783	Signor, PubMed	1, 2
MEK	RAF	Positive; phosphorylation	8413257	Signor, KEGG, PubMed	1, 2
MKK3_6	TAK1	Positive; phosphorylation	21902831	Signor, KEGG, PubMed	1, 2
MKK4_7	TAK1	Positive; phosphorylation	9278437	Signor, KEGG, PubMed	1, 2
MLH1	MYC	Positive; upregulation	15814658	PubMed	2
MMR	MLH1	Positive; upregulation	9500552	Signor, PubMed	2

MSK	ERK	Positive; phosphorylation	16868029	PubMed	1, 2
	MAPK14	Positive; phosphorylation	10806207	Signor, KEGG, PubMed	1, 2
mTORC1	AKT	Positive; phosphorylation	20138985	Signor, KEGG, PubMed	1, 2
	PRAS40	Negative; dissociation	-	KEGG	1, 2
	RHEB	Positive; upregulation	19222999	Signor, KEGG, PubMed	1, 2
mTORC2	PI3K	Positive; phosphorylation	-	KEGG	1, 2
	S6K	Negative; phosphorylation	-	KEGG	1, 2
MYC	ERK	Positive; phosphorylation	-	KEGG	1, 2
	LEF1	Positive; transcriptional regulation	19653274	Signor, KEGG, PubMed	1, 2
	SMAD3	Negative; transcriptional regulation	11689553	Signor, PubMed	1, 2
NER	ERCC1_XPF	Positive; incision	30563071	KEGG, PubMed	1, 2
NFkB	IKK	Positive; phosphorylation	19609947	Signor, KEGG, PubMed	1, 2
	MSK	Positive; phosphorylation	17183360	Signor, PubMed	1, 2

NLK	TAK1	Positive; phosphorylation	12482967	Signor, KEGG, PubMed	1, 2
Non_rep_dam	BER	Negative; repairs damage when functional	-	-	2
	DSB	Positive; induces non-repairable damage in the absence of repair pathway(s)	-	-	1, 2
	HR	Negative; repairs damage when functional	-	-	1, 2
	ICL	Positive; induces non-repairable damage in the absence of repair pathway(s)	-	-	1, 2
	MMR	Negative; repairs damage when functional	-	-	2
	NER	Negative; repairs damage when functional	-	-	1, 2
	ROS	Positive; induces non-repairable damage in the absence of repair pathway(s)	-	-	2
Overall_phenotype	Apoptosis	Positive	-	-	1, 2
	Growth_arrest	Positive	-	-	1, 2

	G1_S_transition	Positive	-	-	1, 2
Oxaliplatin	-	-	-	-	1, 2
p16	Non_rep_dam	Positive; upregulation	32182711, 26385091	PubMed	2
p21	AKT	Negative; phosphorylation	16982699	Signor, KEGG, PubMed	1, 2
	FOXO	Positive; transcriptional regulation	-	KEGG	1, 2
	MYC	Negative; transcriptional regulation	12835716	Signor, PubMed	1, 2
	p53	Positive; transcriptional regulation	21524151	Signor, KEGG, PubMed	1, 2
p27	AKT	Negative; phosphorylation	12042314	Signor, KEGG, PubMed	1, 2
	FOXO	Positive; transcriptional regulation	21440011	Signor, KEGG, PubMed	1, 2
	MYC	Negative; transcriptional regulation	12835716	Signor, PubMed	1, 2
p53	ATM	Positive; phosphorylation	11875057	Signor, KEGG, PubMed	1, 2
	ATR	Positive; phosphorylation	11865061	Signor, KEGG, PubMed	1, 2

	CHEK1	Positive; phosphorylation	15659650	Signor, KEGG, PubMed	1, 2
	CHEK2	Positive; phosphorylation	10673501	Signor, KEGG, PubMed	1, 2
	MDM2	Negative; ubiquitination	23150757	Signor, KEGG, PubMed	1, 2
	Non_rep_dam	Positive; upregulation	31835405	PubMed	1, 2
PARP	CASP3	Negative; cleavage	11907276	Signor, KEGG, PubMed	1, 2
	DSB	Positive; sensing/detection of DNA damage	31273204	PubMed	1, 2
	ROS		25460733	PubMed	2
	SSB		26626479	Signor, PubMed	1, 2
PDK1	PI3K	Positive; activation	20517722	KEGG, PubMed	1, 2
PI3K	GAB1	Positive; binding	11043767	Signor, KEGG, PubMed	1, 2
	IRS1	Positive; activation	20966354	Signor, KEGG, PubMed	1, 2
	PTEN	Negative; dephosphorylation	18794881	Signor, KEGG, PubMed	1, 2
	RAS	Positive; binding	21779497	Signor, KEGG, PubMed	1, 2
PRAS40	AKT	Negative; phosphorylation	12524439	Signor, KEGG, PubMed	1, 2

PTEN	GSK3	Negative; phosphorylation	16107342	Signor, PubMed	1, 2
	IKK	Negative; binding	-	KEGG	1, 2
	NFkB	Negative; suppression	14729949	PubMed	1, 2
RAF	RAS	Positive; binding	21779497	Signor, KEGG, PubMed	1, 2
RAS	SOS	Positive; binding	11560935	Signor, KEGG, PubMed	1, 2
RB1	CDK2_CycE	Negative; phosphorylation	9139732	Signor, KEGG, PubMed	1, 2
	CDK4_6_CycD	Negative; phosphorylation	15809340	Signor, KEGG, PubMed	1, 2
RHEB	TSC1_2	Negative; GTPase-activation	15340059	Signor, KEGG, PubMed	1, 2
RIP1	TRAF2	Positive; binding	10795740, 16953224	Signor, KEGG, PubMed	1, 2
ROS	TNFR	Positive; increases production	20203691	PubMed	2
RSK	ERK	Positive; phosphorylation	9687510	Signor, KEGG, PubMed	1, 2
S6K	mTORC1	Positive; phosphorylation	10567431	Signor, KEGG, PubMed	1, 2

	PDK1	Positive; phosphorylation	9445476	Signor, KEGG, PubMed	1, 2
SHC	PTEN	Negative; phosphorylation-inhibiting	9832564	PubMed	1, 2
SMAD3	JUN	Negative; binding	10871633	Signor, PubMed	1, 2
SOS	GRB2	Positive; binding	10570290	Signor, KEGG, PubMed	1, 2
SSB	Fluorouracil	Positive; induces damage	30316683	PubMed	1, 2
TAK1	RIP1	Positive; binding	21133840, 16953224	Signor, KEGG, PubMed	1, 2
TNF	NFkB	Positive; transcriptional regulation	20219869, 16953224	Signor, PubMed	1, 2
TNFR	TNF	Positive; binding	10634209, 16953224	Signor, KEGG, PubMed	1, 2
TRADD	TNFR	Positive; binding	11502070, 16953224	Signor, KEGG, PubMed	1, 2
TRAF2	TRADD	Positive; binding	14585074, 16953224	Signor, KEGG, PubMed	1, 2
TSC1_2	AKT	Negative; phosphorylation	-	KEGG	1, 2
	ERK	Negative; phosphorylation	15851026	Signor, KEGG, PubMed	1, 2
	GSK3	Positive; phosphorylation	-	KEGG	1, 2

	RSK	Negative; phosphorylation	15342917	Signor, KEGG, PubMed	1, 2

Table S3. List of model nodes, effector nodes and associated logical formulas describing the conditions for node activity in the initial (1) and adjusted models (2), respectively.

**Node**	**Effector node(s)**	**Logical formula (1)**	**Logical formula (2)**
AKT	mTORC2 (+), PDK1 (+), PI3K (+)	PI3K | PDK1 | mTORC2	PI3K | PDK1 | mTORC2
AP1	FOS (+), JUN (+)	FOS | JUN	FOS | JUN
APAF1	CYCS (+)	CYCS	CYCS
Apoptosis	CASP3 (+)	CASP3	CASP3
ATM	DSB (+)	DSB	DSB
ATR	ICL (+), SSB (+)	SSB | ICL	SSB | ICL
BAD	AKT (-), RSK (-)	!(RSK | AKT)	!(RSK | AKT)
BAX	BCL2 (-), p53 (+)	p53 & !BCL2	p53 & !BCL2
BCL2	BAD (-), ERK (+), NFkB (+)	(ERK | NFkB) & !BAD	(ERK | NFkB) & !BAD
BER	PARP (+)	PARP	PARP
BRCA	ATM (+), CHEK2 (+), MEK (+), MYC (+), PI3K (+), ROS (+)	ATM | CHEK2	((ATM | CHEK2) & MEK & MYC & PI3K) | (ROS & MEK & MYC & PI3K)
CASP3	CASP8 (+), CASP9 (+)	CASP9 | CASP8	CASP9 | CASP8

CASP8	CFLAR (-), FADD (+)	FADD & !CFLAR	FADD & !CFLAR
CASP9	APAF1 (+)	APAF1	APAF1
CDC25A	CHEK1 (-), CHEK2 (-), E2F (+), MYC (+)	(MYC | E2F) & !(CHEK1 | CHEK2)	(MYC | E2F) & !(CHEK1 | CHEK2)
CDK2_CycE	E2F (+), CDC25A (+), p16 (-), p21 (-), p27 (-)	(E2F | CDC25A) & !(p27 | p21)	(E2F | CDC25A) & !(p27 | p21 | p16)
CDK4_6_CycD	AP1 (+), CDC25A (+), mTORC1 (+), p16 (-), p21 (-)	(AP1 | CDC25A) & !p21	mTORC1 & (AP1 | CDC25A) & !(p21 | p16)
CFLAR	NFkB (+)	NFkB	NFkB
CHEK1	ATR (+)	ATR	ATR
CHEK2	ATM (+)	ATM	ATM
CYCS	BAX (+)	BAX	BAX
DSB	BER (-), Fluorouracil (+), SSB (+)	Fluorouracil | (SSB & !BER)	Fluorouracil | (SSB & !BER)
E2F	CDK4_6_CycD (+), RB1 (-)	!RB1	CDK4_6_CycD & !RB1
ERCC1_XPF	ICL (+)	ICL	ICL
ERK	MEK (+)	MEK	MEK

FADD	TRADD (+)	TRADD	TRADD
Fluorouracil	-	Input	Input
FOS	ERK (+)	ERK	ERK
FOXO	AKT (-), IKK (-), NLK (-)	!(AKT | NLK | IKK)	!(AKT | NLK | IKK)
G1_S_transition	CDK2_CycE (+)	CDK2_CycE	CDK2_CycE
GAB1	GRB2 (+)	GRB2	GRB2
GRB2	SHC (+), IRS1 (+)	SHC | IRS1	SHC | IRS1
Growth_arrest	CDK2_CycE (-)	!CDK2_CycE	!CDK2_CycE
GSK3	AKT (-)	!AKT	!AKT
HR	BRCA (+)	BRCA	BRCA
ICL	Oxaliplatin (+)	Oxaliplatin	Oxaliplatin
IKK	AKT (+), TAK1 (+)	TAK1 | AKT	TAK1 | AKT
IRS1	IKK (-), JNK (-), S6K (-)	!(S6K | JNK | IKK)	!(S6K | JNK | IKK)
JNK	MKK4_7 (+)	MKK4_7	MKK4_7
JUN	JNK (+)	JNK	JNK

LEF1	NLK (-)	!NLK	!NLK
MAPK14	MKK3_6 (+)	MKK3_6	MKK3_6
MDM2	AKT (+), ATM (-), ATR (-)	AKT & !(ATM | ATR)	AKT & !(ATM | ATR)
MEK	RAF (+)	RAF	RAF
MKK3_6	TAK1 (+)	TAK1	TAK1
MKK4_7	TAK1 (+)	TAK1	TAK1
MLH1	MYC (+)	-	MYC
MMR	MLH1 (+)	-	MLH1
MSK	ERK (+), MAPK14 (+)	ERK | MAPK14	ERK | MAPK14
mTORC1	AKT (+), PRAS40 (-), RHEB (+)	(AKT | RHEB) & !PRAS40	AKT | RHEB | !PRAS40
mTORC2	PI3K (+), S6K (-)	PI3K & !S6K	PI3K & !S6K
MYC	ERK (+), LEF1 (+), SMAD3 (-)	(ERK | LEF1) & !SMAD3	(ERK | LEF1) & !SMAD3
NER	ERCC1_XPF (+)	ERCC1_XPF	ERCC1_XPF
NFkB	IKK (+), MSK (+)	IKK | MSK	IKK | MSK

NLK	TAK1 (+)	TAK1	TAK1
Non_rep_dam	BER (-), DSB (+), HR (-), ICL (+), MMR (-), NER (-), ROS (+)	(ICL & !NER) | (DSB & !HR)	(ICL & !NER) | (DSB & !HR) | (ROS & !(MMR | BER | HR))
Overall_phenotype	Apoptosis (+), Growth_arrest (+), G1_S_transition (+)	3: G1_S_transition & !(Apoptosis | Growth_arrest)	3: G1_S_transition & !(Apoptosis | Growth_arrest)
		2: Growth_arrest & !(Apoptosis | G1_S_transition)	2: Growth_arrest & !(Apoptosis | G1_S_transition)
		1: Apoptosis & !G1_S_transition	1: Apoptosis & !G1_S_transition
		0: !(Apoptosis | Growth_arrest | G1_S_transition)	0: !(Apoptosis | Growth_arrest | G1_S_transition)
Oxaliplatin	-	Input	Input
p16	Non_rep_dam (+)	-	Non_rep_dam
p21	AKT (-), FOXO (+), MYC (-), p53 (+)	(FOXO | p53) & !(AKT | MYC)	(FOXO | p53) & !(AKT | MYC)
p27	AKT (-), FOXO (+), MYC (-)	FOXO & !(AKT | MYC)	FOXO & !(AKT | MYC)
p53	ATM (+), ATR (+), CHEK1 (+), CHEK2 (+), MDM2 (-), Non_rep_dam (+)	(ATM | ATR | CHEK2 | CHEK1 | Non_rep_dam) & !MDM2	(ATM | ATR | CHEK2 | CHEK1 | Non_rep_dam) & !MDM2

PARP	CASP3 (-), DSB (+), ROS (+), SSB (+)	(SSB | DSB) & !CASP3	(SSB | DSB | ROS) & !CASP3
PDK1	PI3K (+)	PI3K	PI3K
PI3K	GAB1 (+), IRS1 (+), PTEN (-), RAS (+)	(RAS | IRS1 | GAB1) & !PTEN	(RAS | IRS1 | GAB1) & !PTEN
PRAS40	AKT (-)	!AKT	!AKT
PTEN	GSK3 (-), IKK (-), NFkB (-)	!(GSK3 | NFkB | IKK)	!(GSK3 | NFkB | IKK)
RAF	RAS (+)	RAS	RAS
RAS	SOS (+)	SOS	SOS
RB1	CDK2_CycE (-), CDK4_6_CycD (-)	!(CDK4_6_CycD | CDK2_CycE)	!(CDK4_6_CycD | CDK2_CycE)
RHEB	TSC1_2 (-)	!TSC1_2	!TSC1_2
RIP1	TRAF2 (+)	TRAF2	TRAF2
ROS	TNFR (+)	-	TNFR
RSK	ERK (+)	ERK	ERK
S6K	mTORC1 (+), PDK1 (+)	PDK1 | mTORC1	PDK1 | mTORC1
SHC	PTEN (-)	!PTEN	!PTEN

SMAD3	JUN (-)	!JUN	!JUN
SOS	GRB2 (+)	GRB2	GRB2
SSB	Fluorouracil (+)	Fluorouracil	Fluorouracil
TAK1	RIP1 (+)	RIP1	RIP1
TNF	NFkB (+)	NFkB	NFkB
TNFR	TNF (+)	TNF	TNF
TRADD	TNFR (+)	TNFR	TNFR
TRAF2	TRADD (+)	TRADD	TRADD
TSC1_2	AKT (-), ERK (-), GSK3 (+), RSK (-)	GSK3 & !(AKT | ERK | RSK)	GSK3 & !(AKT | ERK | RSK)

Table S4. List of model adjustments. *Node* and *Interaction* indicate addition of nodes and interactions, respectively. *Logical formula* indicates addition or adjustment of a logical formula.

**Update order**		**Adjustment(s)**	**Adjustment type**
1	a)	CDK4_6_CycD --> E2F	Interaction
	b)	E2F = CDK4_6_CycD &! RB1	Logical formula
2	a)	mTORC1 --> CDK4_6_CycD	Interaction
	b)	CDK4_6_CycD = (mTORC1|AP1|CDC25A) &! p21	Logical formula
3	-	CDK4_6_CycD = mTORC1|AP1|CDC25A|!p21	Logical formula
4	-	CDK4_6_CycD = mTORC1 & (AP1|CDC25A) & !p21	Logical formula
5	-	mTORC1 = AKT | RHEB | !PRAS40	Logical formula
6	a)	p16	Node
	b)	Non_rep_dam --> p16	Interaction
	c)	p16 --| CDK4_6_CycD	Interaction
	d)	p16 --| CDK2_CycE	Interaction
	e)	p16 = Non_rep_dam	Logical formula
	f)	CDK4_6_CycD = mTORC1 & (AP1 | CDC25A) & !(p21 | p16)	Logical formula
	g)	CDK2_CycE = (E2F | CDC25A) & !(p27 | p21 | p16)	Logical formula
7	a)	MEK --> BRCA	Interaction
	b)	BRCA = ATM | CHEK2 | MEK	Logical formula
8	-	BRCA = (ATM | CHEK2) & MEK	Logical formula
9	a)	MYC --> BRCA	Interaction
	b)	BRCA = (ATM | CHEK2) & (MEK | MYC)	Logical formula
10	-	BRCA = (ATM | CHEK2) & (MEK & MYC)	Logical formula

11	a)	ROS	Node
	b)	MLH1	Node
	c)	MMR	Node
	d)	TNFR --> ROS	Interaction
	e)	ROS --> Non_rep_dam	Interaction
	f)	BER --| Non_rep_dam	Interaction
	g)	MMR --| Non_rep_dam	Interaction
	h)	MYC --> MLH1	Interaction
	i)	MLH1 --> MMR	Interaction
	k)	ROS --> BRCA	Interaction
	l)	ROS --> PARP	Interaction
	m)	ROS = TNFR	Logical formula
	n)	Non_rep_dam = (ICL & !NER) | (DSB & !HR) | (ROS & !(MMR | BER | HR))	Logical formula
	o)	MLH1 = MYC	Logical formula
	p)	MMR = MLH1	Logical formula
	q)	BRCA = ((ATM | CHEK2) & MEK & MYC) | (ROS & MEK & MYC)	Logical formula
	r)	PARP = (SSB | DSB | ROS) &! CASP3	Logical formula
12	a)	PI3K --> BRCA	Interaction
	b)	BRCA = ((ATM | CHEK2) & ((MEK & MYC) | PI3K)) | (ROS & ((MEK & MYC) | PI3K))	Logical formula
13	-	BRCA = ((ATM | CHEK2) & MEK & MYC & PI3K) | (ROS & MEK & MYC & PI3K)	Logical formula

Table S5. Drug combinations with documented synergistic effect (HSA excess ≤ -0.7) in HCT-116 cells (Folkesson et al., 2020).

**Drug combination**	**HSA excess**
PD - PAL	-2.551
5FU - PAL	-2.187
PI - PD	-2.170
PI - 5Z	-1.627
5Z - PAL	-1.530
PD - 5FU	-1.470
5Z - 5FU	-1.272
OXA - PAL	-1.217
PD - OLA	-1.127
PI - 5FU	-1.110
OLA - 5Z	-0.863
PAL – OLA	-0.860
PI – OLA	-0.800

Table S6. List of in vitro perturbations tested in the reference screen (Folkesson et al., 2020) and their associated in silico perturbations in the initial (1) and adjusted (2) models, respectively.

#	**In vitro perturbation**	**In silico perturbation (1)**	**In silico perturbation (2)**
1	Baseline (un-perturbed)	Oxaliplatin KO, Fluorouracil KO	Oxaliplatin KO, Fluorouracil KO, MLH1 KO
2	PI	PI3K KO, Oxaliplatin KO, Fluorouracil KO	PI3K KO, Oxaliplatin KO, Fluorouracil KO, MLH1 KO
3	PD	MEK KO, Oxaliplatin KO, Fluorouracil KO	MEK KO, Oxaliplatin KO, Fluorouracil KO, MLH1 KO
4	PAL	CDK4_6_CycD KO, Oxaliplatin KO, Fluorouracil KO	CDK4_6_CycD KO, Oxaliplatin KO, Fluorouracil KO, MLH1 KO
5	OXA	Oxaliplatin E1, Fluorouracil KO	Oxaliplatin E1, Fluorouracil KO, MLH1 KO
6	OLA	PARP KO, Oxaliplatin KO, Fluorouracil KO	PARP KO, Oxaliplatin KO, Fluorouracil KO, MLH1 KO
7	5Z	TAK1 KO, Oxaliplatin KO, Fluorouracil KO	TAK1 KO, Oxaliplatin KO, Fluorouracil KO, MLH1 KO
8	5FU	Oxaliplatin KO, Fluorouracil E1	Oxaliplatin KO, Fluorouracil E1, MLH1 KO
9	PI-PD	PI3K KO, MEK KO, Oxaliplatin KO, Fluorouracil KO	PI3K KO, MEK KO, Oxaliplatin KO, Fluorouracil KO, MLH1 KO
10	PI-PAL	PI3K KO, CDK4_6_CycD KO, Oxaliplatin KO, Fluorouracil KO	PI3K KO, CDK4_6_CycD KO, Oxaliplatin KO, Fluorouracil KO, MLH1 KO
11	PI-OXA	PI3K KO, Oxaliplatin E1, Fluorouracil KO	PI3K KO, Oxaliplatin E1, Fluorouracil KO, MLH1 KO
12	PI-OLA	PI3K KO, PARP KO, Oxaliplatin KO, Fluorouracil KO	PI3K KO, PARP KO, Oxaliplatin KO, Fluorouracil KO, MLH1 KO
13	PI-5Z	PI3K KO, TAK1 KO, Oxaliplatin KO, Fluorouracil KO	PI3K KO, TAK1 KO, Oxaliplatin KO, Fluorouracil KO, MLH1 KO
14	PI-5FU	PI3K KO, Oxaliplatin KO, Fluorouracil E1	PI3K KO, Oxaliplatin KO, Fluorouracil E1, MLH1 KO

15	PD-PAL	MEK KO, CDK4_6_CycD KO, Oxaliplatin KO, Fluorouracil KO	MEK KO, CDK4_6_CycD KO, Oxaliplatin KO, Fluorouracil KO, MLH1 KO
16	PD-OXA	MEK KO, Oxaliplatin E1, Fluorouracil KO	MEK KO, Oxaliplatin E1, Fluorouracil KO, MLH1 KO
17	PD-OLA	MEK KO, PARP KO, Oxaliplatin KO, Fluorouracil KO	MEK KO, PARP KO, Oxaliplatin KO, Fluorouracil KO, MLH1 KO
18	PD-5Z	MEK KO, TAK1 KO, Oxaliplatin KO, Fluorouracil KO	MEK KO, TAK1 KO, Oxaliplatin KO, Fluorouracil KO, MLH1 KO
19	PD-5FU	MEK KO, Oxaliplatin KO, Fluorouracil E1	MEK KO, Oxaliplatin KO, Fluorouracil E1, MLH1 KO
20	PAL-OLA	CDK4_6_CycD KO, PARP KO, Oxaliplatin KO, Fluorouracil KO	CDK4_6_CycD KO, PARP KO, Oxaliplatin KO, Fluorouracil KO, MLH1 KO
21	OXA-PAL	CDK4_6_CycD KO, Oxaliplatin E1, Fluorouracil KO	CDK4_6_CycD KO, Oxaliplatin E1, Fluorouracil KO, MLH1 KO
22	OXA-5FU	Oxaliplatin E1, Fluorouracil E1	Oxaliplatin E1, Fluorouracil E1, MLH1 KO
23	OLA-OXA	PARP KO, Oxaliplatin E1, Fluorouracil KO	PARP KO, Oxaliplatin E1, Fluorouracil KO, MLH1 KO
24	OLA-5Z	PARP KO, TAK1 KO, Oxaliplatin KO, Fluorouracil KO	PARP KO, TAK1 KO, Oxaliplatin KO, Fluorouracil KO, MLH1 KO
25	OLA-5FU	PARP KO, Oxaliplatin KO, Fluorouracil E1	PARP KO, Oxaliplatin KO, Fluorouracil E1, MLH1 KO
26	5Z-PAL	TAK1 KO, CDK4_6_CycD KO, Oxaliplatin KO, Fluorouracil KO	TAK1 KO, CDK4_6_CycD KO, Oxaliplatin KO, Fluorouracil KO, MLH1 KO
27	5Z-OXA	TAK1 KO, Oxaliplatin E1, Fluorouracil KO	TAK1 KO, Oxaliplatin E1, Fluorouracil KO, MLH1 KO
28	5Z-5FU	TAK1 KO, Oxaliplatin KO, Fluorouracil E1	TAK1 KO, Oxaliplatin KO, Fluorouracil E1, MLH1 KO
29	5FU-PAL	CDK4_6_CycD KO, Oxaliplatin KO, Fluorouracil E1	CDK4_6_CycD KO, Oxaliplatin KO, Fluorouracil E1, MLH1 KO

Table S7. Synergy ranking (based on summed HSA excess) per drug combination in selected ray design (concentrations given in Table M2, Supplementary file 2: Materials and Methods) and full matrix design. The analysis was performed using data from the screen by Folkesson et al. (Folkesson et al., 2020) Only data from the HCT-116 cell line were considered.

Combination	Synergy ranking (summed HSA excess)	
	**Ray design**	**Matrix design**
PD+PI	1	1
5FU+PD	2	2
5FU+5Z	3	3
5FU+PI	4	5
OLA+PI	5	7
5Z+OLA	6	6
OLA+PD	7	4
OXA+PD	8	9
5FU+OLA	9	8
OXA+PI	10	10

Table S8. Coefficient of variation (CV) per readout and biological replicate in HCT-116 cells.

	**Replicate 1**		**Replicate 2**	
	**Viability**	**Confluency**	**Viability**	**Confluency**
**CV (%) per readout**	8.10	5.49	8.65	6.07

Table S9. Results from in silico tests of third-order drug combinations. *See Figshare (https://figshare.com/s/2795463950dfe7e1e804).*

Table S10. Results from in silico tests of fourth-order drug combinations. *See Figshare (https://figshare.com/s/2795463950dfe7e1e804).*

Table S11. Results from statistical testing of relative viability in the high-throughput validation screen. For each dose of indicated third-order combinations (“Combination” column) relative viability was compared to the relative viability of the most effective underlying component (“Compared to” column) using a two-sided Student’s t-test. P-values < 0.05 were considered statistically significant (*). For each comparison all technical replicates from all biological replicates were considered.

Combination	Dose step	Compared to	p-value	Significant
PI+PD+5-FU	1	PI+PD	0.250	
	2	PD+5-FU	0.252	
	3	PD+5-FU	9.52E-06	*
	4	PD+5-FU	9.39E-06	*
PI+PD+OXA	1	PI+PD	0.688	
	2	PI+PD	0.369	
	3	PI+PD	0.000360	*
	4	PI+PD	1.48E-05	*
PI+PD+OLA	1	PD+OLA	0.307	
	2	PD+OLA	0.197	
	3	PI+PD	0.0241	*
	4	PI+PD	0.0105	*
5-FU+5Z+OLA	1	OLA+5-FU	0.165	
	2	5-FU+5Z	0.00204	*
	3	5-FU+5Z	0.0643	
	4	5-FU+5Z	0.000478	*
5-FU+PI+OLA	1	PI+5-FU	0.425	
	2	OLA+5-FU	0.0339	*

	3	PI+5-FU	0.654	
	4	PI+5-FU	0.0763	
5-FU+PD+OLA	1	PD+5-FU	0.00408	*
	2	PD+5-FU	6.82E-05	*
	3	PD+5-FU	0.0257	*
	4	PD+5-FU	0.926	

Table S12. Results from statistical testing of relative viability in the low-throughput validation screen. For each dose of indicated higher-order combinations ("Combination" column) relative viability was compared to the relative viability of the most effective underlying component ("Compared to" column) using a two-sided Student's t-test. P-values < 0.05 were considered statistically significant (*). For each comparison all technical replicates from all biological replicates were considered.

Combination	Dose step	Compared to	p-value	Significant
OXA+5-FU+PI	1	OXA+PI	0.257	
	2	5-FU+PI	0.615	
	3	5-FU+PI	0.780	
OXA+5-FU+PD	1	OXA+PD	0.154	
	2	OXA+PD	0.762	
	3	5-FU+PD	0.810	
OXA+PI+PD	1	PI+PD	0.600	
	2	PI+PD	0.633	
	3	PI+PD	0.000825	*
5-FU+PI+PD	1	PI+PD	0.730	
	2	PI+PD	0.292	
	3	PI+PD	0.0535	
OXA+5-FU+ PI+PD	1	PI+PD	0.774	
	2	5-FU+PI+PD	0.261	
	3	OXA+PI+PD[1]	0.0336	*

[1] The effect of OXA+PI+PD is significantly stronger compared to the effect of OXA+5-FU+PI+PD

1.4 References

Bakkenist, C. J., Lee, J. J., and Schmitz, J. C. (2018). ATM Is Required for the Repair of Oxaliplatin-Induced DNA Damage in Colorectal Cancer. *Clinical Colorectal Cancer* 17, 255–257. doi: 10.1016/j.clcc.2018.09.001.

Chen, Y., Xu, J., Borowicz, S., Collins, C., Huo, D., and Olopade, O. I. (2011). c-Myc activates BRCA1 gene expression through distal promoter elements in breast cancer cells. *BMC Cancer* 11, 246. doi: 10.1186/1471-2407-11-246.

Folkesson, E., Niederdorfer, B., Nakstad, V. T., Thommesen, L., Klinkenberg, G., Lægreid, A., et al. (2020). High-throughput screening reveals higher synergistic effect of MEK inhibitor combinations in colon cancer spheroids. *Sci Rep* 10, 11574. doi: 10.1038/s41598-020-68441-0.

Foucquier, J., and Guedj, M. (2015). Analysis of drug combinations: current methodological landscape. *Pharmacol Res Perspect* 3, e00149. doi: 10.1002/prp2.149.

Ibrahim, Y. H., García-García, C., Serra, V., He, L., Torres-Lockhart, K., Prat, A., et al. (2012). PI3K Inhibition Impairs BRCA1/2 Expression and Sensitizes BRCA-Proficient Triple-Negative Breast Cancer to PARP Inhibition. *Cancer Discovery* 2, 1036–1047. doi: 10.1158/2159-8290.CD-11-0348.

Rehman, F. L., Lord, C. J., and Ashworth, A. (2012). The Promise of Combining Inhibition of PI3K and PARP as Cancer Therapy. *Cancer Discovery* 2, 982–984. doi: 10.1158/2159-8290.CD-12-0433.

Rose, M., Burgess, J. T., O'Byrne, K., Richard, D. J., and Bolderson, E. (2020). PARP Inhibitors: Clinical Relevance, Mechanisms of Action and Tumor Resistance. *Front. Cell Dev. Biol.* 8, 564601. doi: 10.3389/fcell.2020.564601.

Vena, F., Jia, R., Esfandiari, A., Garcia-Gomez, J. J., Rodriguez-Justo, M., Ma, J., et al. (2018). MEK inhibition leads to BRCA2 downregulation and sensitization to DNA damaging agents in pancreas and ovarian cancer models. *Oncotarget* 9, 11592–11603. doi: 10.18632/oncotarget.24294.

2 Supplementary file 2: Materials and Methods

Table M1. Material components (cells, drugs, reagents etc.) used in validation screens.

**Product name**	**Reference number**	**Manufacturer**
HCT-116	CVCL_0291	National Cancer Institute (NCI)
Fetal Bovine Serum	F7524	Sigma Aldrich
L-Glutamine solution	G7513	Sigma Aldrich
Penicillin-Streptomycin	15140-122	ThermoFisher Scientific
RPMI 1640, no glutamine	31870-025	ThermoFisher Scientific
TrypLE Express (1X)	12605-010	Gibco
Masterblock, 96 well	780271	Greiner Bio-one GmbH
Trypan Blue stain 0.4%	T10282	Life Technologies
Assay Plate, 384 well	3764	Corning
Dimethyl Sulfoxide	D2650	Sigma Aldrich
Digitonin	D141	Sigma Aldrich
5-Fluorouracil (5FU)	F6627	Sigma Aldrich
Olaparib	S1060	Selleckchem
Oxaliplatin	S1224	Selleckchem
5Z-7-oxozeaenol	ALX-380-267-M005	Enzo Life Sciences
PD0325901	PZ0162	Sigma Aldrich
PI-103	S1038	Selleckchem
Staurosporine	S4400	Sigma Aldrich
D4+ Dispensehead Casettes	F0L60A	Hewlet-Packard (HP)

T8+ Dispensehead Casettes	F0L59A	Hewlet-Packard (HP)
CellTiter-Glo	G9243	Promega

Table M2. Drugs and drug concentrations per dose step included in the high-throughput validation screen.

Type	Associated third order combination(s)	Single drug/ combination abbreviation	Concentration (μM) per dose step[1]			
			1	2	3	4
Single drug	1-4	5FU	2.5	5	10	20
	3	5Z	0.01	0.05	0.25	1.25
	1-3, 6	OLA	2.5	5	10	20
	5	OXA	0.05	0.25	1.25	2.5
	1, 4-6	PD	0.01	0.05	0.25	1.25
	2, 4-6	PI	0.01	0.05	0.25	1.25
Pairwise combination	1-3	5FU+OLA	2.5+2.5	5+5	10+10	20+20
	1, 4	5FU+PD	2.5+0.01	5+0.05	10+0.25	20+1.25
	1, 6	OLA+PD	2.5+0.01	5+0.05	10+0.25	20+1.25
	2, 4	5FU+PI	2.5+0.01	5+0.05	10+0.25	20+1.25
	2, 6	OLA+PI	2.5+0.01	5+0.05	10+0.25	20+1.25
	3	5FU+5Z	2.5+0.01	5+0.05	10+0.25	20+1.25
	3	OLA+5Z	2.5+0.01	5+0.05	10+0.25	20+1.25
	4-6	PD+PI	0.01+0.01	0.05+0.05	0.25+0.25	1.25+1.25
	5	OXA+PD	0.05+0.01	0.25+0.05	1.25+0.25	2.5+1.25
	5	OXA+PI	0.05+0.01	0.25+0.05	1.25+0.25	2.5+1.25
Third-order combination	1	5FU+OLA+PD	2.5+2.5+0.01	5+5+0.05	10+10+0.25	20+20+1.25
	2	5FU+OLA+PI	2.5+2.5+0.01	5+5+0.05	10+10+0.25	20+20+1.25
	3	5FU+OLA+5Z	2.5+2.5+0.01	5+5+0.05	10+10+0.25	20+20+1.25
	4	5FU+PD+PI	2.5+0.01+0.01	5+0.05+0.05	10+0.25+0.25	20+1.25+1.25
	5	OXA+PD+PI	0.05+0.01+0.01	0.25+0.05+0.05	1.25+0.25+0.25	2.5+1.25+1.25

	6	OLA+PD+PI	2.5+0.01+0.01	5+0.05+0.05	10+0.25+0.25	20+1.25+1.25
Controls	-	Digitonin	100	33.333	11.111	3.704
	-	Staurosporine	5	1.67	0.556	0.185
	-	DMSO	0.489%			

[1] µg/ml for digitonin

Table M3. Drugs and drug concentrations per dose step included in the low-throughput validation screen.

Type	Single drug/ combination abbreviation	Concentration (µM) per dose step		
		1	2	3
Single drug	5FU	0.6	3	15
	OXA	0.6	3	15
	PD	0.25	1.25	6.25
	PI	0.25	1.25	6.25
Pairwise combination	5FU+OXA	0.6+0.6	3+3	15+15
	5FU+PD	0.6+0.25	3+1.25	15+6.25
	5FU+PI	0.6+0.25	3+1.25	15+6.25
	OXA+PD	0.6+0.25	3+1.25	15+6.25
	OXA+PI	0.6+0.25	3+1.25	15+6.25
	PD+PI	0.25+0.25	1.25+1.25	6.25+6.25
Third-order combination	5FU+OXA+PD	0.6+0.6+0.25	3+3+1.25	15+15+6.25
	5FU+OXA+PI	0.6+0.6+0.25	3+3+1.25	15+15+6.25
	5FU+PD+PI	0.6+0.25+0.25	3+1.25+1.25	15+6.25+6.25
	OXA+PD+PI	0.6+0.25+0.25	3+1.25+1.25	15+6.25+6.25
Fourth order combination	5FU+OXA+PD+PI	0.6+0.6+0.25+0.25	3+3+1.25+1.25	15+15+6.25+6.25
Control	DMSO	0.5%		

Table M4. Image re-analysis settings used in SoftMax Pro 6.5.1. Unless anything else is stated, default settings were used.

**Setting**	**Sub-setting**	**Plate(s)**
Read Modes	Re-analysis	All plates
Image Analysis settings: Find Objects	Method: CellsC<br>Grow by: 3 pixels	0h plates
Image Analysis settings: Find Objects	Method: CellsC<br>Grow by: 5 pixels	24h plates
Image Analysis settings: Find Objects	Method: CellsA<br>Grow by: 5 pixels	48h plates
Image Analysis settings: Select Measurements	Covered Area (%)	All plates
